# ATM-mediated ELL phosphorylation enhances its self-association through increased EAF1 interaction and inhibits global transcription during genotoxic stress

**DOI:** 10.1093/nar/gkac943

**Published:** 2022-10-28

**Authors:** Sujay Pal, Dipika Yadav, Debabrata Biswas

**Affiliations:** Laboratory of Transcription Biology, Molecular Genetics Division, CSIR-Indian Institute of Chemical Biology, 4, Raja S. C. Mullick Road, Kolkata - 32, India; Academy of Scientific and Innovative Research (AcSIR), Ghaziabad 201002, India; Laboratory of Transcription Biology, Molecular Genetics Division, CSIR-Indian Institute of Chemical Biology, 4, Raja S. C. Mullick Road, Kolkata - 32, India; Laboratory of Transcription Biology, Molecular Genetics Division, CSIR-Indian Institute of Chemical Biology, 4, Raja S. C. Mullick Road, Kolkata - 32, India; Academy of Scientific and Innovative Research (AcSIR), Ghaziabad 201002, India

## Abstract

Mammalian cells immediately inhibit transcription upon exposure to genotoxic stress to avoid fatal collision between ongoing transcription and newly recruited DNA repair machineries to protect genomic integrity. However, mechanisms of this early transcriptional inhibition are poorly understood. In this study, we decipher a novel role of human EAF1, a positive regulator of ELL-dependent RNA Polymerase II-mediated transcription *in vitro*, in regulation of temporal inhibition of transcription during genotoxic stress. Our results show that, besides Super Elongation Complex (SEC) and Little Elongation Complex (LEC), human ELL (aka ELL1) also forms a complex with EAF1 alone. Interestingly, contrary to the *in vitro* studies, EAF1 inhibits ELL-dependent RNA polymerase II-mediated transcription of diverse target genes. Mechanistically, we show that intrinsic self-association property of ELL leads to its reduced interaction with other SEC components. EAF1 enhances ELL self-association and thus reduces its interaction with other SEC components leading to transcriptional inhibition. Physiologically, we show that upon exposure to genotoxic stress, ATM-mediated ELL phosphorylation-dependent enhanced EAF1 association results in reduced ELL interaction with other SEC components that lead to global transcriptional inhibition. Thus, we describe an important mechanism of dynamic transcriptional regulation during genotoxic stress involving post-translational modification of a key elongation factor.

## INTRODUCTION

Immediately upon exposure to genotoxic stress, such as DNA-damaging reagents, mammalian cells employ global transcriptional downregulation as survival mechanism (1). Initial transcriptional inhibition is believed to be important for avoiding fatal collision between ongoing transcription as well as newly recruited DNA repair machineries and thus to avoid further DNA damage causing genomic instability (2,3). Several mechanisms have been proposed for global transcriptional inhibition upon exposure to genotoxic stress. Among them, ubiquitylation-mediated degradation of RNA polymerase II (Pol II, hereafter) is believed to be one of the key mechanisms in overall transcriptional downregulation that is also commonly known as ‘mechanisms of last resort’ (4,5). In support of this mechanism, two recent studies have shown key role of genotoxic stress-dependent ubiquitylation of RPB1 (Pol II largest subunit) at K1286 residue in overall transcriptional downregulation as well as recovery afterwards (6,7). Importantly, after release from promoter region, Pol II transcribes the entire coding region with the help of transcription elongation factors that remain associated with Pol II. Therefore, it is quite conceivable that overall functional regulation of elongation factors, upon exposure to DNA damaging reagents, would also play important roles in regulation of transcription during exposure to genotoxic stress.

Among all the transcription elongation factors, regulation by recently described super elongation complex (SEC) has gained immense interests because of its involvement with MLL fusion-mediated acute form of both lymphoid and myeloid leukemia predominantly observed in pediatric patients (8). Human SEC was described as ∼1.5 MDa large multisubunit complex containing AF9, AF9-family protein ENL, AFF1, AFF4, ELL, ELL-associated factors (EAF1 and EAF2) and P-TEFb complex (a heterodimer of CDK9 and CyclinT1) (9–11). With the exception of the P-TEFb complex and EAF1/2, all other members of the SEC are frequently fused with the N-terminus of the MLL to give rise to MLL fusion proteins that in conjunction with wild type MLL give rise to pediatric leukemia (8). Our earlier study has shown a role for p300-mediated acetylation of AFF1 in global transcriptional downregulation within mammalian cells (12). Although the role of ELL in transcriptional restart during recovery stage after exposure to genotoxic stress has been discussed (13), mechanisms of its functional regulation for attaining efficient transcriptional inhibition is completely unknown.

Among all the SEC components, ELL is the only *bona fide* elongation factor that directly stimulates transcription elongation by Pol II *in vitro* (9,14,15). EAF1 (16,17) directly stimulates ELL-mediated elongation activity of Pol II *in vitro* (9,14). However, the role of EAF1 in ELL-mediated target gene expression within mammalian cells is poorly known. A recent study has indicated a negative role of this ELL-interacting protein in transcriptional regulation without providing mechanistic insights (if any) as well as its physiological relevance (18). In this study, we decipher a novel mechanism of functional regulation of ELL in association with EAF1 that plays key roles in transcriptional downregulation during exposure to genotoxic stress for optimal repair of damaged DNA and cell survival.

## MATERIALS AND METHODS

### Plasmids, primers, and antibodies

List of plasmids, primers used for RNA and ChIP analyses by qRT-PCR, oligos used for making shRNA constructs, and details of antibodies used are mentioned in Supplemental Tables S1–S5 in supplemental materials and methods section.

### Cell culture and transfection

HEK293T cell line used in this study was cultured and maintained in DMEM media (Gibco), supplemented with 10% FBS (Gibco) and 1% penicillin–streptomycin (Invitrogen). The cells were grown at 37°C in presence of 5% CO_2_. The Sf9 cells were cultured in Grace's insect media (HiMedia), supplemented with 10% FBS (Invitrogen) and 7 μg/ml gentamycin (Gold Biotechnology) at 26°C. For transfection in mammalian cells, Fugene transfection reagent was used as per manufacturer's protocol. The Sf9 cells were transfected with Cellfectin II reagent (Invitrogen) following manufacturer's instructions to generate baculoviruses expressing target recombinant proteins. For interaction analyses, cells were harvested 48 h post-transfection, unless otherwise mentioned.

### Generation of plasmid constructs

For expression of target proteins within mammalian system, all the constructs were cloned in pcDNA5/FRT/TO vector with respective epitope tags as mentioned. To express proteins tagged with GFP epitope, constructs were cloned into pEGFP-N2 vector. For expression of proteins in Sf9 cells through baculovirus, constructs were cloned in pFASTBac vectors. pET-11d and pET-GST vectors were used to clone His-tagged and GST-tagged constructs respectively for expression in bacterial system. Details of cloning methods, including restriction sites used for cloning, would be available upon request.

### Generation of stable knockdown cells

For stable knockdown, target shRNAs were cloned into lentiviral pLKO.1-puro vector. To generate lentiviruses, the target shRNA constructs (500 ng) were co-transfected with 125 ng pMD2.G (envelope plasmid) and 375 ng pSPAX2 (packaging plasmid) in 50–70% confluent cells in a single well of 6-well plate. The lentiviral supernatant was collected post 72 h of transfection and stored at −80°C for subsequent use. For specific stable knockdown, cells were transduced with 300 μl of respective virus particles in presence of 8 μg/ml polybrene. Twenty-four hours after transduction, cells were subjected to puromycin selection (3 μg/ml). The positively selected cells were subsequently checked for knockdown efficiency by western blotting using target factor-specific antibodies.

### Nuclear extract preparation

Cells were first harvested in PBS and centrifuged at 3K RPM for 5 min to estimate the packed cell volume (PCV). The cell pellet was subsequently resuspended in 2× PCV of NE1 buffer (10 mM Tris–Cl pH 7.3, 1.5 mM MgCl_2_, 10 mM NaCl) containing 0.7 μl/ml β-mercaptoethanol and kept on ice for 15 min. The cell suspension was passaged through a 23-gauge needle for 8–10 times. This was followed by centrifugation at 6K RPM for 5 min at 4°C. The supernatant was discarded and nuclear pellet volume (NPV) was estimated. The nuclear pellet was subsequently resuspended in 2× NPV of pre-chilled NE2 buffer (20 mM Tris–Cl pH 7.3, 1.5 mM MgCl_2_, 20 mM NaCl, 0.2 mM EDTA and 25% glycerol) containing protease inhibitor cocktail (Roche) and 0.7μl/ml β-mercaptoethanol. This was followed by addition of 1× NPV of prechilled NE3 buffer (20 mM Tris–Cl pH 7.3, 1.5 mM MgCl_2_, 1.2 M NaCl, 0.2 mM EDTA, 25% glycerol) containing protease inhibitor cocktail and 0.7 μl/ml β-mercaptoethanol through mild vortexing. The sample is subsequently incubated on ice for 45 min and vortexed intermittently every 3 min for efficient nuclear extraction. The sample was centrifuged at 12K RPM for 20 min at 4°C. The supernatant was collected as nuclear extract and used for experimental analysis.

### Whole cell lysis, immunoprecipitation and western blot analysis

For immunoprecipitation and interaction analyses, respective epitope-tagged proteins were transiently expressed in 293T cells. The cells were harvested post 48 h of transfection, unless otherwise mentioned, and lysed in lysis buffer (20 mM Tris–Cl pH 8, 20% glycerol, 2 mM EDTA, 150 mM KCl), supplemented with 0.1% NP-40, protease inhibitor cocktail, 0.7 μl/ml β-mercaptoethanol and 2 mM PMSF. The lysates over-expressing FLAG- and HA-tagged proteins were incubated with anti-FLAG M2 agarose beads and anti-HA agarose beads respectively for 12 h at 4°C. The immunoprecipitated samples were washed extensively with the lysis buffer supplemented with 0.1% NP-40 and eluted in 1X SDS dye through incubation at 95°C for 8–10 min.

For identifying interacting partners of immunoprecipitated target proteins, the heat denatured samples were subjected to 8–10% SDS-PAGE at 100V for required amount of time. The properly resolved proteins were subsequently transferred to nitrocellulose membrane in transfer buffer containing 10–15% methanol at 100V for 2 h. The membranes were blocked with 5% skim milk (HiMedia) for 1 h at room temperature. The blocked membranes were further incubated with target primary antibodies with appropriate dilutions for 12 h at 4°C. The membranes were subsequently washed three times with TBST (1× TBS + 0.1% Tween 20) followed by incubation with species-specific secondary antibodies. The membranes were further washed thrice and blots were developed in Azure 300 gel imaging system (Azure Biosystems) or iBright imaging system (Thermo Fisher Scientific) using ECL (BioRad).

### Immunoprecipitation of endogenous proteins

The 293T cells were lysed in BC150 buffer (20 mM Tris–Cl pH 8, 20% glycerol, 2 mM EDTA, 150 mM KCl), supplemented with 0.1% NP-40, protease inhibitor cocktail, 0.7 μl/ml β-mercaptoethanol and 2 mM PMSF. The whole cell lysate was subsequently pre-cleared with protein-A agarose beads (Invitrogen) for 2 h at 4°C. In parallel, protein-G magnetic beads were blocked with 1% BSA in BC150 buffer (supplemented with 0.1% NP-40) for 2 h at 4°C. The pre-blocked protein-G magnetic beads were washed three times with BC150 buffer (+0.1% NP-40) and incubated with 2 μg of target-specific antibodies& species-specific IgG as negative control. The pre-cleared cell lysates were subsequently incubated with antibody-bound protein-G magnetic beads for 12 h at 4°C. The beads were subsequently washed thrice with BC150 buffer (+0.1% NP-40) and heat eluted in 1× SDS dye by incubating at 95°C for 8–10 min.

### RNA extraction, reverse transcription and qRT-PCR analyses

Total cellular RNA was extracted using TRIzol reagent (Invitrogen Inc.) following manufacturer's protocol. Subsequently, RNA (500 ng) was reverse transcribed to cDNA using verso cDNA synthesis kit (Thermo Scientific) following manufacturer's protocol. The synthesized cDNA was further diluted 25 times and used as template for subsequent qRT-PCR analysis. qRT-PCR was done using iTaq Universal SYBR Green Supermix (BIORAD) and target specific primers. The relative RNA analysis was done by normalizing the target mRNA expression with expression of actin as internal control.

### Chromatin immunoprecipitation (ChIP) analysis

ChIP analysis was performed following the same protocol as mentioned earlier (19–21). Cells were cross-linked with 1% formaldehyde (Sigma) for 10 min at room temperature (RT). The reaction was stopped by addition of 125 mM glycine (Sigma) for 5 min at RT. The cross-linked cells were washed in ice cold PBS and resuspended in ChIP lysis buffer (0.5% NP-40, 1% Triton X-100, 150 mM NaCl, 20 mM Tris–Cl pH 7.5, 2 mM EDTA), supplemented with protease inhibitor cocktail and incubated on ice for 30 min. The cells were subsequently passaged through a 23-gauge syringe for 8–10 times on ice and centrifuged at 6K RPM for 10 min at 4°C. The nuclear pellet thus obtained was resuspended in ice cold shearing buffer (1% SDS, 50 mM Tris–Cl pH 8, 10 mM EDTA) and sonicated using Bioruptor™ UCD-200 (Diagenode) sonicator for 20 min (30 s pulse on and off). The sonicated sheared sample was subsequently centrifuged at 12K RPM for 20 min at 4°C. The sample was further diluted 10× in ChIP dilution buffer (0.01% SDS, 1.1% Triton X-100, 1.1 mM EDTA, 20 mM Tris–Cl pH 8 and 167 mM NaCl). The diluted sample was first pre-cleared with IgG for 2 h and subsequently incubated with protein-G magnetic beads (Biorad) for 4 h at 4°C. The immunoprecipitation was carried out by incubating the pre-cleared sample with 2 μg of target antibodies for 10–12 h at 4°C. In a parallel set up, protein-G magnetic beads (Biorad) were blocked by incubating with dilution buffer containing salmon sperm DNA (4μg/μl) for 10–12 h at 4°C. The pre-blocked beads were subsequently added to the antibody-bound protein sample and incubated for 2 h at 4°C. The precipitated protein-G magnetic bead-bound proteins were subsequently washed sequentially with low salt buffer (0.1% SDS, 1% Triton X-100, 2 mM EDTA, 20 mM Tris–Cl pH 8, 150 mM NaCl; supplemented with protease inhibitor cocktail), high salt buffer (0.1% SDS, 1% Triton X-100, 2 mM EDTA, 20 mM Tris–Cl pH 8.0, 500 mM NaCl; supplemented with Protease inhibitor cocktail), lithium chloride buffer (0.5 M LiCl, 1% NP-40, 1% deoxycholate, 20 mM Tris–Cl pH 8, 1mM EDTA; supplemented with protease inhibitor cocktail) and finally with TE buffer (10 mM Tris–Cl pH 8, 1 mM EDTA). The immunoprecipitated DNA was subsequently eluted in elution buffer (1% SDS, 0.1 M NaHCO_3_) at room temperature. The eluted sample was reverse cross-linked by incubating at 65°C for 6–10 h in presence of 200mM NaCl. The sample was further treated with proteinase K (Sigma) at 45°C for 45 min. Finally, the immunoprecipitated DNA was purified using QIAquick PCR purification kit (Qiagen) as per manufacturer's protocol. The purified DNA was subsequently used as template for qRT-PCR analysis (BIORAD CFX96™ Real-Time-System) to quantify enrichment of specific factors at indicated regions on target gene.

### Luciferase reporter assay

Gal4-luciferase cell line was used for carrying out luciferase reporter assay (22). This cell line contains a adenovirus major late promoter-driven chromosomally-integrated luciferase reporter gene downstream to five Gal4 binding sites. For experiments, cells were transfected with respective plasmid constructs expressing the target proteins (as indicated) and cells were harvested post 48 h of transfection. The reporter luciferase activity was checked by Dual-Glo® Luciferase Assay System (Promega) using GloMax 20/20 Luminometer (Promega) by following manufacturer's protocol.

### Recombinant protein purification

For expression and purification of GST-tagged proteins (GST-alone and GST-ELL), BL21(DE3) *Escherichia coli* cells were transformed with respective plasmid constructs cloned in pET-GST vectors. The expression of recombinant proteins in bacteria had been induced with 1mM IPTG (GoldBio) at 37°C for 4 h. The cells were subsequently harvested and lysed in lysis buffer (20 mM Tris–Cl pH 8, 300 mM KCl, 2 mM EDTA, 20% glycerol; supplemented with 0.1% NP40, protease inhibitor cocktail, 0.7 μl/ml β-mercaptoethanol and 2 mM PMSF) through sonication for 5 min (at 60% amplitude, with 30 s pulse on and off) on ice. The whole cell lysate was subsequently centrifuged at 12K RPM for 20 min at 4°C. The solubilized fraction (supernatant) was subsequently incubated with glutathione agarose beads (Pierce) for 4 h at 4°C. The beads were washed extensively and stored as bead-bound proteins in BC150 buffer (20 mM Tris–Cl pH 8, 300 mM KCl, 2 mM EDTA, 20% glycerol) at 4°C.

For expression of His-GFP-ELL (cloned in pET-11d vector) in BL21(DE3) *E. coli* cells, the secondary culture was induced at 18°C with 1 mM IPTG (GoldBio) for 15–18 h. The harvested cells were sonicated in lysis buffer (50 mM Na_2_HPO_4_, 300 mM NaCl, 10 mM imidazole, 20% glycerol, pH 8; supplemented with 0.1% NP40, protease inhibitor cocktail, 0.7 μl/ml β-mercaptoethanol and 2 mM PMSF) as mentioned earlier. The solubilized fraction was collected after centrifuging at 12K RPM for 20 min at 4°C and subjected to binding with Ni-NTA beads for 2 h at 4°C. The bead-bound proteins were washed extensively in wash buffer (50 mM Na_2_HPO_4_, 300 mM NaCl, 20 mM imidazole, 20% glycerol, pH 8; supplemented with 0.1% NP40) and eluted in elution buffer (50 mM Na_2_HPO_4_, 300 mM NaCl, 250 mM imidazole, 20% glycerol, pH 8; supplemented with 0.1% NP40).

### Glycerol gradient fractionation

A 4–20% glycerol gradient was prepared and kept overnight at 4°C for uniform gradient formation. Nuclear extract from 293T cells was loaded onto the top of the gradient. The sample was then centrifuged at 40K RPM for 8 h at 4°C. Multiple fractions were carefully collected from the top of the gradients and the proteins were subsequently precipitated by methanol/chloroform precipitation method. The presence of different target proteins in each fractions was identified by western blotting using factor specific antibodies.

### *In vitro* cross-linking assay

Whole cell lysates from 293T cells were incubated with mild concentration (0.03–0.01%) of cross-linking agent glutaraldehyde and incubated for indicated time periods at room temperature. The reactions were then stopped by adding 5× SDS dye and the samples were subsequently prepared by heating at 95°C for 10 min. The cross-linked samples were then subjected to SDS-PAGE followed by western blotting using factor-specific antibodies.

### Native PAGE analysis

The samples for native PAGE analysis were prepared by mixing purified His-GFP-ELL proteins with 4× loading buffer (62.5 mM Tris–Cl pH 6.8, 25% glycerol and 0.01% bromophenol blue) in absence of reducing agent β-mercaptoethanol. The gels were prepared under non-denaturing condition (without SDS)and had been run at 4°C using ice cold Tris-glycine running buffer. 4 μg of BSA was loaded on to the gel as molecular weight marker under native condition.

### Baculovirus expression-based protein complex reconstitution assay

The protein complex reconstitution analyses using baculoviral expression system have been performed by following the same protocol as mentioned earlier (22). Briefly, the Sf9 cells were co-infected with viruses expressing respective target proteins. The cells were harvested post 48 h of infection and lysed in lysis buffer (20 mM Tris–Cl pH 8, 20% glycerol, 2 mM EDTA, 300 mM KCl), supplemented with 0.1% NP-40, protease inhibitor cocktail, 0.7 μl/ml β-mercaptoethanol and 2 mM PMSF. For pull down, antibody-specific agarose beads were used against the epitope tag and incubated for 12 h at 4°C. The bead-bound proteins were washed extensively in lysis buffer supplemented with 0.1% NP-40. The bead-bound proteins were subsequently eluted using 3× FLAG peptides at a concentration of 250 ng/μl. The eluted fractions were further subjected to second round of immunoprecipitations as mentioned in the result section.

### *In vitro* interaction analyses

For *in vitro* interaction analysis, GST-bead-bound proteins (GST-alone, GST-ELL) were incubated with experimental prey proteins (His-GFP-ELL or His-EAF1) in binding buffer (20 mM Tris–Cl pH 8, 20% glycerol, 2 mM EDTA, 150 mM KCl) in presence of BSA (20 ng/μl) and 0.1% NP-40 for 10–12 h at 4°C. The beads were washed with binding buffer, containing 0.1% NP-40, extensively and subsequently heat eluted in 1× SDS loading dye by incubating at 95°C for 8–10 min. The samples were subsequently subjected to SDS-PAGE and western blotting for interaction analyses.

### Immunofluorescence analysis

293T cells were grown on cover slips in 12-well plates and transfected with plasmid constructs expressing FLAG/GFP-tagged respective proteins. The cells were subsequently fixed in 4% paraformaldehyde (Sigma) for 15 min at room temperature. The cells were further washed with PBS once and permeabilized with 0.5% Triton-X for 15 min at room temperature. This is followed by blocking in 1% BSA (Sigma) for 1 h at room temperature. The cells were subsequently incubated with respective primary antibodies (1:1000 dilutions) for 12 h at 4°C. The cells were washed with PBS and incubated with species-specific secondary Alexa-fluor (594) antibodies (1:500 dilutions) for 1 h at room temperature. The cells were washed and the nuclear DNA was stained with hoechst dye (in PBS) for 15 min at RT. The cells were further washed and proceeded for imaging using LSM 800 (ZEISS) confocal microscope. The obtained images were subsequently analyzed using Zen 2.3 lite software.

### Nascent RNA transcription analysis

The nascent RNA transcription analyses were performed using Click-iT™ RNA Alexa Fluor™ 488 Imaging Kit (Invitrogen) following manufacturer's protocol. Like immunofluorescence analysis, cells were seeded on coverslips in 12-well plate and transfected with plasmid constructs expressing FLAG-ELL WT/FLAG-ELL (45–621)/FLAG-ELL TM, respectively. Post 40 h of transfection, the cells were treated with 1 μM of doxorubicin for 2 h. 0.3 mM 5-ethynyl uridine (EU) was added to the media 15 min prior to the completion of doxorubicin treatment. The cells were subsequently fixed, permeabilized and blocked as mentioned for immunofluorescence studies. The permeabilized cells were incubated in Click-iT reaction cocktail for 30 min at RT. The cells were subsequently washed with rinse buffer at RT. The washed cells were further incubated with primary antibody specific for FLAG epitope tag (1:1000) for 12 h at 4°C. The cells were subsequently washed with 1× PBS and incubated with anti-rabbit Alexa Fluor 594 (1:500) at room temperature for 1 h. The cells were washed again with 1× PBS and the nuclear DNA was stained with hoechst dye (in PBS) at room temperature for 15 min and proceeded for imaging using LSM 800 (ZEISS) microscope. The obtained images were subsequently analyzed using Zen 2.3 lite software.

### Colony formation assay

For comparative analysis of colony formation abilities, 1 × 10^4^ respective cells (scramble, ELL KD and EAF1 KD) were seeded in each well of 6-well plate and allowed to grow for 7–10 days to form distinct colonies. The colonies were fixed with fixing solution consisting of methanol and acetic acid (3:1) for 15 min and subsequently stained in 0.5% crystal violet (in methanol) for 15 min at RT. The plates were subsequently washed thoroughly with water to remove excessive background stains and images were captured.

### Cell proliferation assay

293T cells were first transfected with respective plasmid constructs ((FLAG-ELL(WT)/ FLAG-ELL(45–621)/ FLAG-ELL(TM)) as mentioned and were treated with 100nM of doxorubicin for 2 h after 40 h of transfection. Approximately 1 × 10^4^ doxorubicin treated cells were seeded and cells were counted by using hemocytometer on second and fourth day after seeding. For comparative analysis of cell proliferation abilities between scramble and ELL KD cells, ∼1 × 10^4^ cells were seeded and counted on the mentioned days after seeding.

## RESULTS

### ELL regulates SEC-mediated expression of diverse set of genes within mammalian cells

**Figure 1.**
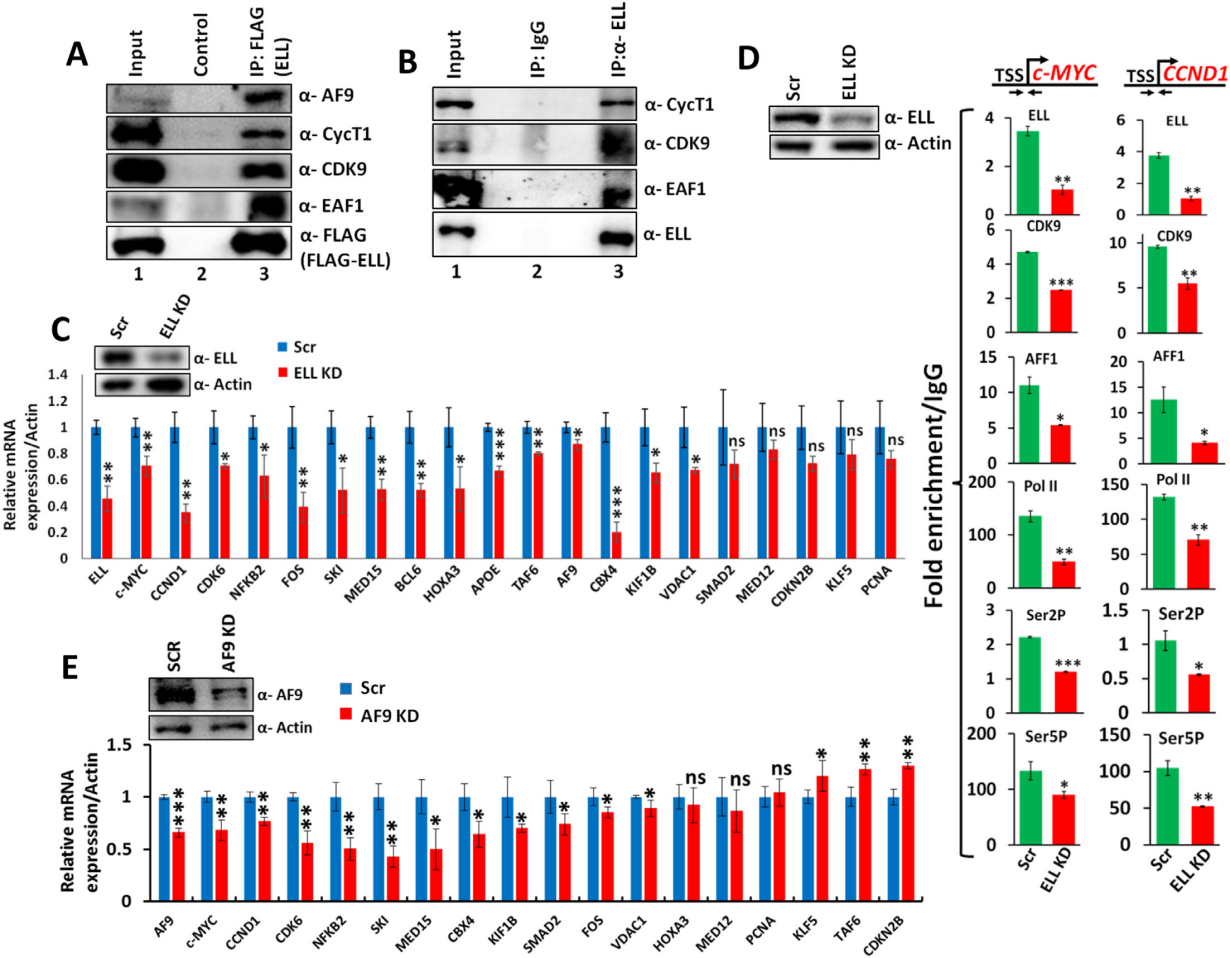
ELL is required for basal level expression of multiple SEC target genes within mammalian cells. (**A**) Immunoblot analysis showing interaction between ectopically-expressed FLAG-ELL and different endogenous SEC components. (**B**) Immunoprecipitation of endogenous ELL showing its interaction with other SEC components within mammalian cells. (**C**) qRT-PCR analyses showing significant downregulation of expression of multiple SEC target genes upon knockdown of ELL as compared to control scramble cells. The relative RNA expressions were analyzed by normalizing the target mRNA expressions with that of actin (as internal control). (**D**) ChIP analyses showing reduced recruitment of SEC components on target genes at the promoter proximal region upon ELL knockdown. IgG was used as control in our experiments for calculating the fold enrichment. (**E**) qRT-PCR analyses showing significant downregulation of expression of multiple SEC target genes upon knockdown of AF9 as compared to control scramble cells. The relative RNA expressions were analyzed by normalizing the target mRNA expressions with that of Actin (as internal control).

Human ELL protein has been shown to be present in multiple different (sub)complexes including SEC and LEC (9,23–25). The ELL-containing LEC has been proposed to play important role in Pol II-mediated snRNA gene transcription (24). Although isoforms of ELL, ELL2 and ELL3 have been described in SEC-mediated regulation of HIV TAT-dependent transcription and enhancer functions in embryonic stem cells respectively (10,26,27), the role of ELL-containing SEC in Pol II-mediated transcription is still controversial. In an effort towards addressing these questions, we initially tested whether ELL would show any functional interaction with other SEC components. Consistent with the idea of ELL regulating SEC-dependent transcription of target genes, we observed ELL interaction with other SEC components including EAF1 within mammalian cells (Figure 1A). Similar interactions were also observed with endogenous ELL when immunoprecipitated using specific antibody (Figure 1B). For addressing these interactions in regulating target gene expression within cells, we generated stable ELL knockdown cells by using shRNA (Figure 1C, upper panel). Subsequent RNA analysis using qRT-PCR showed significant downregulation of expression of diverse target genes including *CCND1* and *c-MYC* (Figure 1C, lower panel) whose expressions are being regulated by SEC components (12,19,20,28,29). This effect is specific since in the same assays, we failed to observe significant effect on expression of few other genes. Subsequent ChIP analyses showed reduced recruitment of majority of SEC components including Pol II at the promoter proximal region of the target *CCND1* and *c-MYC* genes (Figure 1D) and thus suggesting a key role of ELL in regulating recruitment of other SEC components on these target genes. Consistent with a role of ELL in regulation of SEC-mediated target gene expression, we have also observed significant overlap between set of genes that showed downregulation of expression upon knockdown of AF9 (as a representative of SEC components) (Figure 1E) as well as ELL (Figure 1C). Since majority of these genes are involved in proliferation and cell cycle regulation, we have also observed significant reduction of proliferation and colony formation ability upon knockdown of ELL (Supplementary Figure S1A, B). Overall, these results suggest that along with its known function in association with LEC, human ELL also plays key roles in regulating SEC-mediated target gene expression within mammalian cells.

### EAF1 negatively regulates ELL-dependent expression of chromosomally-integrated GAL4-luciferase reporter gene

The role of EAF1 and EAF2 in SEC-mediated transcriptional regulation within mammalian cells is still not known. Initial studies suggested a role for these factors in stimulating ELL-mediated, Pol II-dependent transcription *in vitro* (9,14). A recent study has shown a negative role of these factors in SEC-mediated HIV TAT-dependent transcription within mammalian cells without providing any mechanistic insights (18). As part of our initial understanding of these regulations, we observed interaction of SEC components with EAF1 by immunoprecipitation analyses both in the context of its ectopic (Supplementary Figure S2A) as well as endogenous expression (Supplementary Figure S2B).

Next, for our initial understanding of role of EAF1/2 in regulating ELL-mediated target gene expression *in vivo*, we employed a cell-based chromosomally-integrated five copies of GAL4 DNA binding-containing adenovirus major late promoter-driven reporter luciferase gene expression analysis (Supplementary Figure S2C, upper panel) (22,30). Consistent with a direct role of ELL in regulating transcription elongation, ectopic expression of ELL as GAL4 fusion (GAL4-ELL), strongly increased reporter gene activity (Supplementary Figure S2C, lower panel, GAL4-ELL alone). However, to our surprise, over-expression of EAF1 drastically reduced ELL-mediated reporter gene activation in a dose-dependent manner (Supplementary Figure S2C lower panel, compare GAL4-ELL and GAL4-ELL + EAF1 lanes). The overall effect is not a result of reduced ELL expression since our blotting analysis showed no negative effect of EAF1 on ELL expression (Supplementary Figure S2C middle panel). In fact, over-expression of EAF1 showed to increase the expression of GAL4-ELL (Supplementary Figure S2C, middle panel, compare lane 2 versus lanes 4–6). The seemingly different effect of EAF1 on ELL-mediated target gene expression in a cell-based assay when compared to *in vitro* assays (9,14), led us to further investigate mechanistic insights into this overall regulation. Our co-immunoprecipitation analysis upon overexpression of EAF1 clearly showed its negative role on ELL interaction with other SEC components upon its binding (Supplementary Figure S2D, compare lane 5 versus lane 6). Thus, we conclude that the EAF1 protein competes with other SEC components for their binding to ELL and thus potentially results in transcriptional downregulation.

Next, we performed chromatin immunoprecipitation (ChIP) analyses for addressing factor recruitment at promoter proximal region of the target reporter gene. As shown in Supplementary Figure S2E, and consistent with a positive role of ELL in reporter gene activation, we observed significant increase in GAL4-ELL binding (as monitored by ELL ChIP in comparison with empty vector control) along with other SEC components as monitored by CDK9 and AFF1 ChIP (Supplementary Figure S2E, CDK9 and AFF1 panels). Consistent with enhanced recruitment of CDK9 and AFF1, we also observed a marked increase in the Ser2 and Ser5 phosphorylated form of Pol II indicating active transcriptional activity (Pol II Ser2P and Pol II Ser5P panels). Interestingly, concomitant over-expression of EAF1 and its binding significantly reduced SEC recruitment at the promoter proximal region without reducing Pol II recruitment. Reduced CDK9 recruitment also resulted in reduced presence of phosphorylated Ser2 and Ser5 form of Pol II. Further, consistent with a role of P-TEFb-mediated Ser2 and Ser5 phosphorylation in releasing paused Pol II, we also observed an increase in Pol II at promoter proximal region indicating presence of paused Pol II at this region. Interestingly, although in the presence of EAF1, ectopic expression of GAL4-ELL is increased (Supplementary Figure S2C, ELL panel), we have consistently observed decreased recruitment of ELL (that represents both endogenous ELL as well as GAL4-ELL) in presence of EAF1 and thus further indicates a negative role of EAF1 in recruiting ELL at the promoter proximal region. Thus, based on the above results, we conclude that human EAF1 negatively regulates ELL association with other SEC components resulting in their reduced recruitment and subsequent expression of chromosomally-integrated target reporter gene.

### Differential effect of EAF2 in regulating ELL association with other SEC components and reporter gene expression

Next, we aimed to check the effect of EAF2, a homolog of EAF1, on ELL-mediated activation of chromosomally-integrated reporter gene expression. Unlike EAF1, that interacts at both N and C-termini, EAF2 has been shown to interact only at the N-terminus of ELL (16). Interestingly, contrary to the effect of EAF1, over-expression of EAF2 failed to show any effect on ELL interaction with other SEC components (Supplementary Figure S2F, compare lane 2 versus lane 3). In fact, we consistently observed a modest increase in ELL interaction with other SEC components upon binding to EAF2. Further, and consistent with this observation, we also observed a dose-dependent increase in ELL-mediated reporter gene expression upon increasing expression of EAF2 (Supplementary Figure S2G). This result is different from the effect that was reported using HIV TAT-based reporter gene expression (18). The difference in assay systems being used (naked DNA versus chromosomally-integrated reporter gene as well as adenovirus major late promoter versus HIV TAT) could possibly explain this overall discrepancy. Nevertheless, based on our interaction analyses and assay systems, we conclude that EAF1 and EAF2 protein differentially affect ELL association with other SEC components and thus target reporter gene expression within mammalian cells.

### EAF1 negatively regulates ELL-dependent expression of native target genes

**Figure 2.**
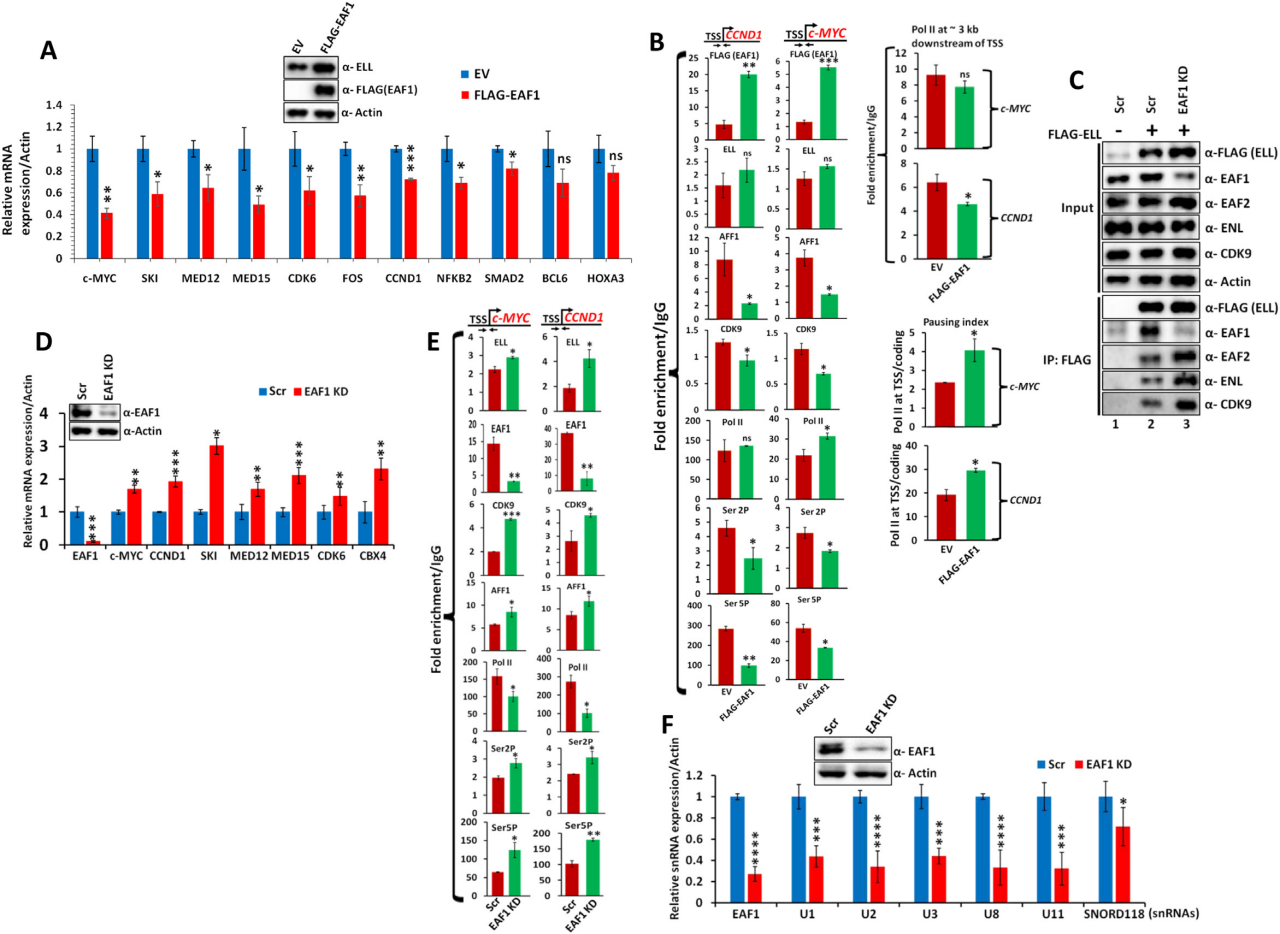
EAF1 negatively regulates expression of native ELL target genes within mammalian cells. (**A**) qRT-PCR analyses showing negative effect of EAF1 overexpression on expression of native ELL target genes within 293T cells. 293T cells were transfected with plasmid constructs expressing empty vector (EV) and FLAG-EAF1 respectively. The relative RNA analyses were analyzed by normalizing the target mRNA expressions with that of Actin (as internal control). The inset panels show overexpression of EAF1 at protein level. (**B**) The left panel represents ChIP analyses showing negative effect of EAF1 overexpression on recruitment of different SEC factors on ELL target genes at the promoter proximal regions. The fold enrichment of different factors on target region had been normalized with that of IgG control. The top right panel shows relative amount of Pol II at the indicated coding regions of target *CCND1* and *c-MYC* genes. The bottom right panel shows pausing index of Pol II (a ratio of Pol II at promoter proximal region/coding region) at the target *CCND1* and *c-MYC* genes. (**C**) Immunoblot analysis showing enhanced interaction between ELL and other SEC components upon EAF1 knockdown. (**D**) qRT-PCR analyses showing enhanced expression of different ELL native target genes in EAF1 knockdown cells. The relative RNA analyses were analyzed by normalizing the target mRNA expressions with that of Actin (as internal control). (**E**) ChIP analyses showing increased recruitment of different SEC components on ELL target genes at the promoter proximal regions upon EAF1 knockdown. The fold enrichment of different factors on target region had been normalized with that of IgG control. (**F**) qRT-PCR analyses showing reduced expression of several snRNA target genes upon EAF1 knockdown within mammalian cells. The relative RNA analyses were analyzed by normalizing the target snRNA expressions with that of Actin (as internal control). For Figures 1 and 2, all of our qRT-PCR analyses for ChIP and RNA analyses, the error bar represents mean ± SD and statistical analyses were performed using one/two tailed Student's *t* test wherein * denotes *P* ≤ 0.05, ** denotes *P* ≤ 0.01, *** denotes *P* ≤ 0.001, and ns denotes ‘not significant’. Data represents a minimum of n = 2 biological replicates and three PCR replicates for each sample.

Based on our results using chromosomally-integrated reporter gene expression, we addressed whether similar mechanisms of action would also be observed for ELL-mediated transcription of native target genes within mammalian cells. In our initial assays, we addressed whether over-expression of EAF1 (Figure 2A, upper panels) would show any effect on expression of ELL-target genes (Figure 1C). Interestingly, significant number of these genes showed reduced expression upon over-expression of EAF1 as observed through qRT-PCR analyses (Figure 2A, lower panels). Subsequent ChIP analyses at target *CCND1* and *c-MYC* genes further confirmed that upon overexpression of EAF1 and its recruitment at the promoter proximal region, ELL recruitment remained somewhat similar in both the genes that we have tested (Figure 2B, ELL panel). However, recruitment of other SEC components such as AFF1 and CDK9 were significantly impaired (Figure 2B, AFF1 and CDK9 panels). This reduced P-TEFb recruitment resulted in reduced level of Pol II CTD Ser2 and Ser5 phosphorylation (Figure 2B, Ser2P, and Ser5P panels). Further normalization of phosphorylated Ser2 and Ser5 form with that of total amount of Pol II being present at the promoter proximal region showed significant reduction of these two marks (Supplementary Figure S3A). Consistent with reduced presence of Ser2 and Ser5 phosphorylation, we also observed reduced release of Pol II from this region (Figure 2B, Pol II panel). Consistent with this reduced release, we also observed reduced presence of Pol II at the coding region of these genes (Figure 2B, upper right panels). Further, pausing index (a ratio of Pol II being present at the promoter proximal region/coding region) analyses showed increased pausing of Pol II upon overexpression of EAF1 on these two native ELL target genes that we have tested (Figure 2B, lower right panels). However, similar overexpression of EAF2 failed to show significant decrease in the expression of majority of these target genes within mammalian cells (Supplementary Figure S3B) and further showing an evidence of differential effect of these two homologous proteins in controlling expression of target genes within mammalian cells.

Next, to avoid the artifacts associated with overexpression of EAF1 and conclusions drawn from the associated data, we knocked down EAF1 and tested its effect on mRNA expression of native target genes of ELL. Interestingly, as shown in Figure 2C, contrary to its effect of overexpression, knockdown of EAF1 resulted in marked increase in ELL interaction with other SEC components in our immunoprecipitation analysis (compare lane 2 versus lane 3). Consistent with this increased interaction, we also observed significant increase in mRNA expression of ELL-target genes that we have tested (Figure 2D). Further, we have also observed increased SEC component recruitment at the target *CCND1* and *c-MYC* genes upon EAF1 knockdown (Figure 2E) by ChIP analyses. Enhanced recruitment of SEC components such as P-TEFb complex and AFF1 also results in increased presence of Pol II CTD Ser2 and Ser5 phosphorylation (Figure 2E, Ser2P and Ser5P panels and Supplementary Figure S3C) resulting in enhanced release of Pol II from the promoter proximal region (Figure 2E, Pol II panel). However, contrary to the effect of EAF1, we have failed to observe any marked effect in ELL interaction with SEC components upon EAF2 knockdown (Supplementary Figure S3D). Thus, based on all these evidences, we conclude that human EAF1 and EAF2 differentially affect the expression of ELL-target genes within mammalian cells, in which, the EAF1 reduces association of ELL with other SEC components and their subsequent recruitment resulting in reduced target gene expression, whereas, EAF2 fails to do so.

Further, along with an effect of ELL interaction with SEC components, we have also observed similar effect on ELL interaction with LEC component (ICE1) upon over-expression of EAF1 (Supplementary Figure S3E). Consistent with this interaction, we have also observed reduced expression of multiple snRNA genes that we have tested upon overexpression of EAF1 (Supplementary Figure S3F). However, unlike its effect on expression of SEC target genes, knockdown of EAF1 showed reduced expression of snRNA gene expression (Figure 2F). Since expression of snRNA genes require critical presence of LEC (24), a knockdown of EAF1 critically affects the overall LEC abundance and thus affect the expression of snRNA genes. Since EAF1 has shown a differential effect on overall ELL-mediated SEC-dependent target gene expression which is different from the earlier reported *in vitro* studies (9,14), our subsequent studies are aimed at detailed molecular understanding of role of EAF1 in functional regulation of ELL-containing SEC and its implication in regulation of cellular processes.

### ELL-containing SEC does not include EAF1

**Figure 3.**
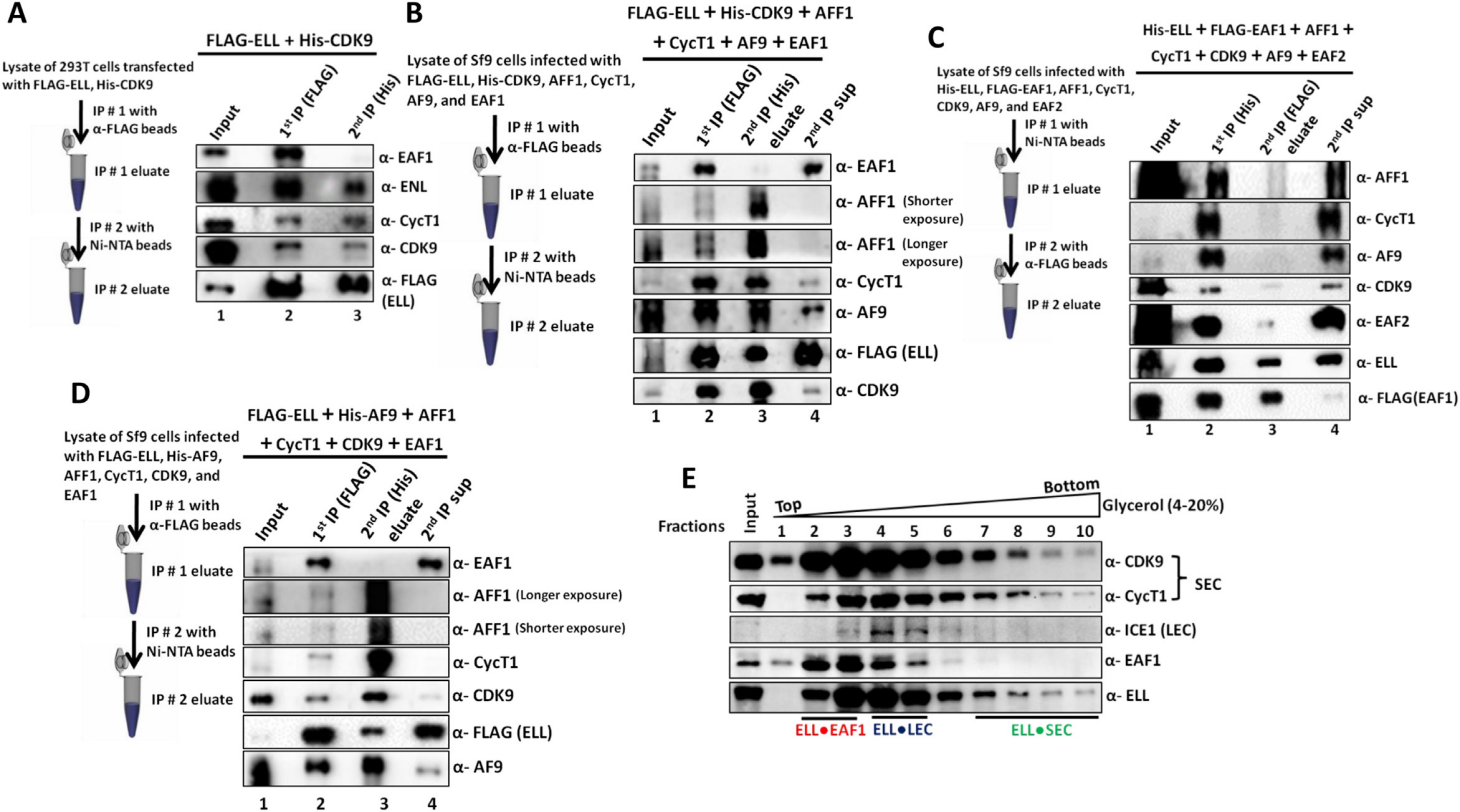
EAF1-containing ELL complex does not associate with other common SEC components. (**A**) Immunoblot analysis showing the absence of EAF1 in ELL•CDK9•SEC in 293T cells. The left panel shows the experimental strategy for tandem affinity purification used for this experiment and the right panel represents the immunoblot analyses to identify the interacting proteins using factor-specific antibodies in each step. (**B**) Immunoblot analysis showing the absence of EAF1 in ELL•CDK9•SEC through reconstitution of protein complex using baculovirus-mediated expression of indicated target recombinant proteins in heterologous Sf9 cells. The left panel shows the experimental strategy for tandem affinity purification used for this assay and right panel represents the immunoblot analyses to identify the interacting proteins using factor-specific antibodies in each step. (**C**) Immunoblot analysis showing formation of a separate ELL•EAF1 complex other than ELL•SEC through reconstitution of protein complex using baculovirus-mediated expression of indicated target recombinant proteins in heterologous Sf9 cells. The left panel shows the experimental strategy for tandem affinity purification used for this assay and right panel represents the immunoblot analyses to identify the interacting proteins using factor-specific antibodies in each step. (**D**) Immunoblot analysis showing the absence of EAF1 in ELL•AF9•SEC through reconstitution of protein complex using baculovirus-mediated expression of indicated target recombinant proteins in heterologous Sf9 cells. The left panel shows the experimental strategy for tandem affinity purification used for this assay and right panel represents the immunoblot analyses to identify the interacting proteins using factor-specific antibodies in each step. (**E**) Glycerol gradient-based separation of protein complex present in the nuclear extract of 293T cells. Nuclear extract was loaded onto a 4–20% glycerol gradient and was separated by centrifugation. Fractions were collected and individual fractions were tested for presence of indicated proteins by western blotting.

The apparent differential effect of EAF1 and EAF2 on expression of ELL-target genes and the inhibitory effect of EAF1 on the association between ELL and other SEC components raised the possibility that EAF1 may not be a part of the ELL-containing SEC (ELL•SEC) that is involved in transcriptional activation. To address this, we took the advantage of tandem affinity purification for purifying ELL-containing SEC from mammalian system using the experimental approach as mentioned in Figure 3A (left panel). As shown in Figure 3A, the first round of IP using FLAG-ELL showed presence of all the tested SEC components including ENL, CyclinT1 and CDK9 along with EAF1 (lane 2). However, and most interestingly, the second round of IP with His-CDK9 (from the first IP eluate), which represents ELL•CDK9•SEC, retained association with ENL, CyclinT1, and CDK9, but failed to show the presence of EAF1 (Figure 3A, lane 3). To provide direct evidence and to rule out any indirect effect of the presence of other proteins within mammalian cells in the overall complex formation, we used protein complex reconstitution assays through baculovirus-mediated expression of these proteins in heterologous Sf9 cells. In our initial analysis, we co-infected Sf9 cells with baculoviruses expressing FLAG-ELL, His-CDK9 and other SEC factors (as mentioned in Figure 3B) along with EAF1. The first round of IP with FLAG-ELL showed presence of all the target SEC factors along with EAF1 (Figure 3B, lane 2). However, and consistent with our analysis from mammalian cells, the second round of IP using His-CDK9 showed presence of all the SEC components except EAF1 (Figure 3B, lane 3). Interestingly, the presence of EAF1 and ELL in the supernatant obtained after the second round of IP (lane 4) clearly suggested formation of a mini ELL•EAF1 complex within this heterologous system that is distinct from ELL•CDK9•SEC as observed in the second round of IP analysis. Thus, in this study, we are reporting presence of mini ELL•EAF1 complex that is distinct from earlier reported SEC and LEC (24,25,31,32).

For providing further evidence of the existence of the ELL•EAF1 complex, we have performed another protein complex reconstitution analysis in Sf9 cells using the strategy as mentioned in Figure 3C (left panel) using His-ELL and FLAG-EAF1 as epitope-tagged proteins along with other untagged SEC components. As expected, in the first round of IP using His-ELL, we observed the presence of all the SEC components along with EAF1 (Figure 3C, lane 2). Interestingly, and consistent with our earlier observation, the second round of IP using FLAG-EAF1 showed the presence of only ELL•EAF1 complex without other SEC components, thus representing only ELL•EAF1 subcomplex in this population (Figure 3C, lane 3). Further and most importantly, parallel blotting analyses showed the presence of all other SEC components in the supernatant obtained after second round of IP, thus providing additional evidence of EAF1 complex formation only with ELL and not with other SEC components including EAF2 (Figure 3C, lane 4). Our further analysis using FLAG-ELL and another representative SEC component, AF9, also showed similar results as obtained through using FLAG-ELL and His-CDK9 (Figure 3D).

Next, to provide an evidence of existence of a separate ELL•EAF1 complex within mammalian cells, we performed glycerol gradient-mediated separation of protein complexes using nuclear extract obtained from 293T cells. As shown in Figure 3E, the top fractions that represents the low molecular weight proteins, clearly showed the presence of ELL•EAF1 complex (fractions 2 and 3) which did not show presence of LEC components, such as ICE1. Although these fractions showed presence of P-TEFb complex components, these possibly represent free form of P-TEFb which has similar molecular weight as that of ELL•EAF1. The ELL-associated P-TEFb complex (representing the SEC) is observed in the later fractions (fractions 7–10). Interestingly, these fractions failed to show presence of EAF1 in the protein complexes. Based on these evidences, we conclude that human ELL forms a separate complex with EAF1 (ELL•EAF1) that is distinct from the ones described for SEC and LEC. Of note, the EAF1 interaction with other SEC components (Supplementary Figure S2A-B) represents ELL-independent interaction and can have other functional implications than regulating ELL-mediated transcription within mammalian cells.

### ELL is a self-associated protein which forms a separate complex with EAF1 than ELL•SEC

**Figure 4.**
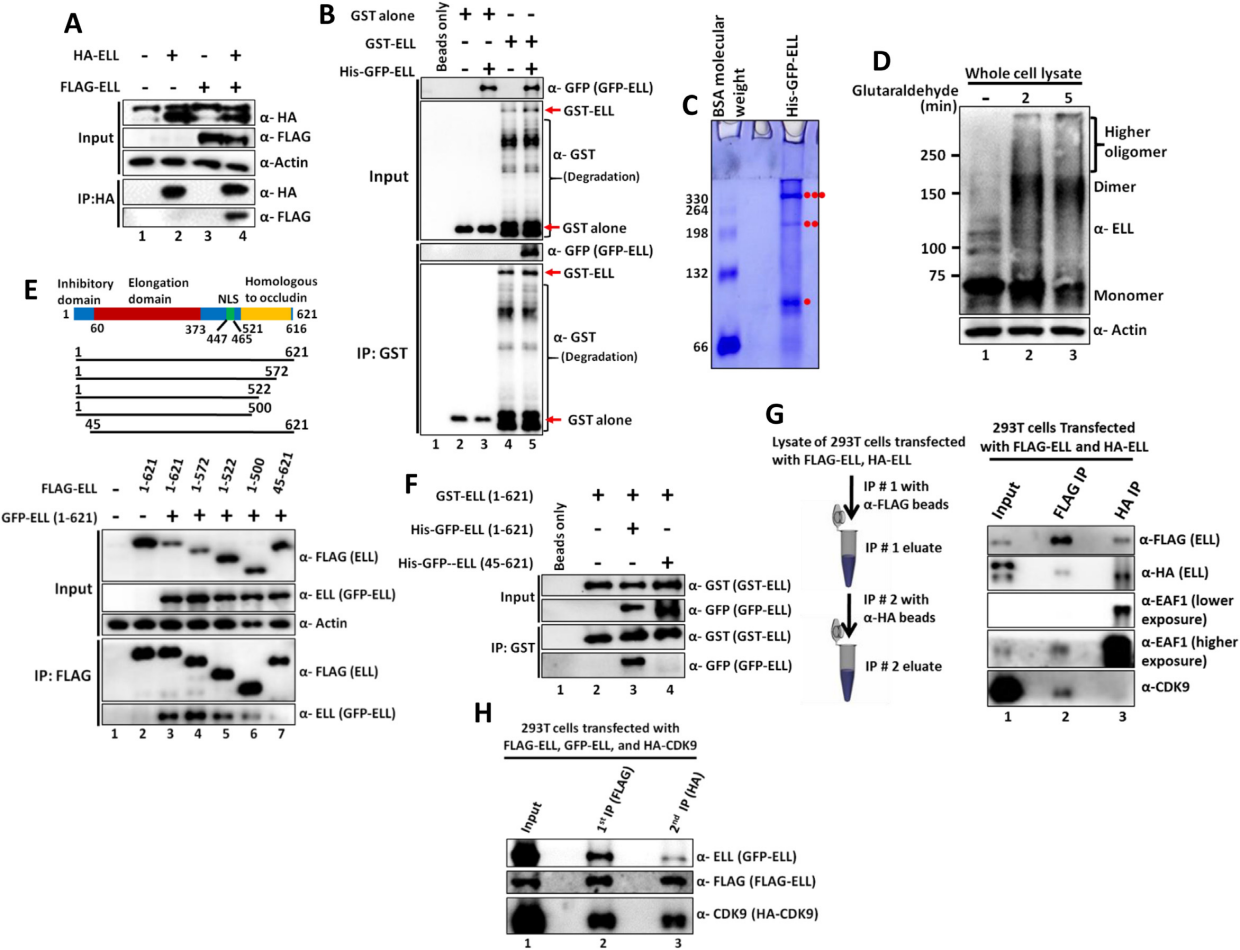
ELL is a self-associated protein and self-associated ELL does not contain other SEC components. (**A**) Immunoblot analysis showing self-association between HA- and FLAG-tagged ELL within mammalian cells. (**B**) Immunoblot analysis showing self-association between purified recombinant ELL proteins at physiological salt concentration (150mM NaCl) *in vitro*. (**C**) Native-PAGE analyses and subsequent coomassie staining showing formation of multimeric ELL complexes by purified recombinant ELL protein. Presence of monomer, dimer and trimer species are indicated by •, ••, and ••• respectively. (**D**) Glutaraldehyde cross-linking based assay showing presence of monomer, dimer and higher oligomeric species of ELL within whole cell lysate of mammalian 293T cells. (**E**) Immunoblot analysis showing self-association ability of different ELL domains within mammalian cells. (**F**) Immunoblot analysis showing defective self-association in vitro between ELL molecules upon deletion of N-terminal 44 amino acids. (**G**) Immunoblot analysis showing the presence of EAF1 with self-associated ELL that does not interact with other SEC components. The left panel shows the experimental strategy for tandem affinity purification used for this experiment and the right panel represents the immunoblot analyses to identify the interacting proteins using factor-specific antibodies in each step. (**H**) Immunoblot analysis showing markedly reduced presence of self-associated ELL in ELL•CDK9 complex in 293T cells by tandem affinity purification strategy.

Our biochemical data suggests existence of three different ELL containing complexes, ELL•SEC, ELL•LEC, and ELL•EAF1 (Figure 3). We further focused on functional regulation by ELL•SEC and ELL•EAF1 complexes and its implications on regulation of cellular processes. What are the underlying molecular mechanism(s) that regulate(s) formation of these different complexes? Protein self-association (dimerization and/or oligomerization) has been shown to play a determining role in dynamic regulation of multiple complex formation. For example, our previous study has shown that ZMYND8, as a dimeric protein, associates with the transcriptional activator PTEF-b whereas the monomeric protein interacts with the repressor NuRD complex (22). Based on this understanding, we wanted to check the possibility of ELL being a self-associated protein. To test this, we co-transfected 293T cells with plasmids expressing FLAG-ELL and HA-ELL. Subsequent IP and blotting analysis confirmed interaction between HA-ELL and FLAG-ELL (Figure 4A, lane 4) and thus confirmed self-association between ELL proteins within mammalian cells. To provide direct evidence of this self-interaction, we purified recombinant ELL proteins as GST- and His-GFP-tagged through their expression in bacteria (Supplementary Figure S4A). *In vitro* interaction analysis using these purified proteins clearly showed direct interaction between GST-ELL and His-GFP-ELL (Figure 4B, lane 5). This interaction is specific since, in a parallel analysis, purified GST protein alone failed to show any interaction with His-GFP-ELL (Figure 4B, lane 3). These data clearly demonstrate self-association between ELL proteins both *in vitro* and *in vivo* within mammalian cells. Further, along with similar lines of evidence, we have also observed strong colocalization of GFP-ELL and FLAG-ELL (indicating self-association) when they were co-expressed within mammalian 293T cells (Supplementary Figure S4B).

For deeper understanding the nature of ELL self-association and resultant complex formation, we subjected our purified recombinant His-GFP-ELL in native PAGE analyses. Consistent with our observation of ELL self-association, we also observed formation of multiple ELL self-associated complexes including dimer, trimer, as well as higher oligomer (Figure 4C). Consistent with this, glycerol gradient analyses with purified recombinant His-GFP ELL showed predominant presence of monomeric ELL in the early fractions (containing low molecular weight proteins) and higher oligomeric species in the later fractions (Supplementary Figure S4C). Formation of the self-associated ELL complex is critically dependent on the presence of reducing reagents (β-mercaptoethanol) in the sample buffer since addition of lower amount of reducing reagent facilitated formation of higher oligomeric self-associated ELL complex (Supplementary Figure S4D). Consistent with our observation with purified recombinant ELL protein, we also observed formation of monomer, dimer as well as higher oligomeric ELL complex in the whole cell lysate when cross-linked with glutaraldehyde and subsequently analyzed by SDS-PAGE (Figure 4D, lanes 2 and 3). All these analyses clearly showed that human ELL is a self-associated protein and this self-association leads to formation of several ELL associated species including monomer, dimer, trimer as well as higher oligomeric species.

To further investigate the molecular mechanisms of ELL self-association, we performed domain analysis of ELL to identify the specific domain within ELL that would be involved for this self-association. We co-transfected several FLAG-tagged ELL deletion constructs with GFP-ELL within 293T cells (Figure 4E, upper panel). Subsequent IP using FLAG epitope tag showed that a) ELL self-association is significantly dependent on the presence of the C-terminal 501–621 fragment since losing this region markedly impairs self-association (Figure 4E lower panel, compare lane 3 versus lane 6) and b) most importantly, deletion of the N-terminal 44 amino acids almost abolished ELL self-association (Figure 4E, compare lane 3 versus lane 7). Consistent with a role of N-terminal 44 amino acids in ELL self-association, we failed to observe ELL self-association when a purified ELL fragment (45–621) (Supplementary Figure S4E) was used in our self-association assay *in vitro* (Figure 4F, compare lane 3 versus lane 4). Further, the N-terminal 44 amino acids were described as inhibitory domain by an earlier analysis (33) and no functions were ascribed to the C-terminal occludin homology domain that shows presence of coiled-coil region (Supplementary Figure S4F) by publicly available prediction software paircoil2 (34). Since coiled-coil region is important for protein self-association as observed by our earlier study (22), this region, in conjunction with N-terminal 44 amino acids, could play important roles in overall ELL self-association and its functional regulation (see below).

To further identify the importance of ELL self-association and protein complex formation, and thus its functional regulation, we purified self-associated ELL from 293T cells co-expressing FLAG- and HA-tagged ELL through tandem affinity purification as shown in Figure 4G (left panel). This purified self-associated ELL failed to show the presence of key SEC component such as CDK9 (Figure 4G, lane 3). However, interestingly, the same assay showed strong presence of EAF1 with the self-associated ELL (Figure 4G, lane 3) suggesting preferential association of EAF1 with self-associated ELL. Further, using similar analyses, the ELL-containing CDK9 complex (representing ELL•SEC) showed marked reduced presence of GFP-ELL in the second round of immunoprecipitated sample (Figure 4H, compare lane 2 versus lane 3). Thus, these results clearly indicate that N- and C-terminal domain-mediated self-association of ELL regulates its association with other SEC components in which, the self-association inhibits SEC association and concomitantly shows enhanced association with EAF1.

### EAF1 increases while EAF2 decreases ELL self-association

**Figure 5.**
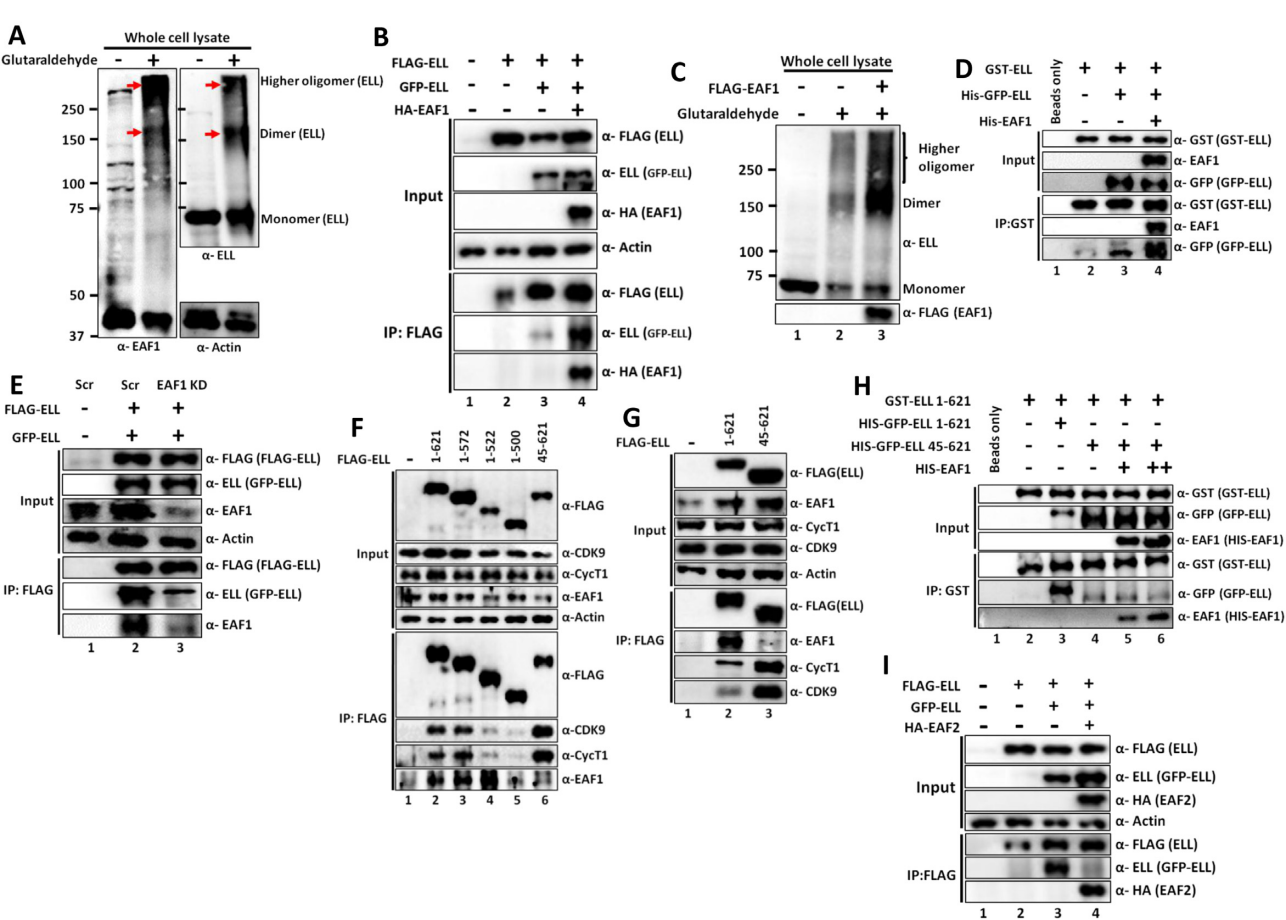
EAF1 enhances ELL self-association both in vitro and in vivo within mammalian cells. (**A**) Immunoblot analysis showing presence of EAF1 in the oligomeric (dimer and above) species of ELL, but not in monomer. (**B**) Immunoblot analysis showing enhanced ELL self-association upon co-expression of EAF1 within mammalian cells. (**C**) Glutaraldehyde cross-linking based assay showing presence of enhanced formation of higher oligomeric species of ELL within whole cell lysate of mammalian 293T cells upon over-expression of EAF1. (**D**) Immunoblot analysis showing enhanced ELL self-association in presence of EAF1, using purified recombinant proteins *in vitro*. (**E**) Immunoblot analysis showing reduced self-association among ELL proteins upon EAF1 knockdown in 293T cells. (**F**) Immunoblot analysis showing the abilities of ELL domains to interact with EAF1 and other SEC components. (**G**) Immunoblot analysis showing the ELL domain (45–621)that is defective of EAF1 interaction, shows enhanced association with other SEC components. (**H**) Immunoblot analysis showing failure of EAF1 protein to enhance ELL self-association between full-length (1–621) and N-terminal deletion fragment (45–621) in *in vitro* self-association assay. (**I**) Immunoblot analysis showing reduced ELL self-association upon overexpression of EAF2 within mammalian cells.

Based on the observation that self-associated ELL contains EAF1, but not other SEC components (Figure 4), and that EAF1 association decreases ELL interaction with other SEC components (Supplementary Figure S2D), we hypothesized that human EAF1 may enhance ELL self-association and thus could form the underlying mechanism of its inhibitory effect. Our initial analysis using whole cell lysate clearly showed the presence of EAF1 in the higher oligomeric ELL species and not in the monomer (Figure 5A). Consistent with this observation, cell-based studies showed enhanced ELL self-assembly upon co-expression of EAF1 (Figure 5B, compare lane 3 versus lane 4). Further, over-expression of EAF1 also promoted higher oligomeric ELL complex formation within mammalian cells (Figure 5C, compare lane2 versus lane 3). For showing a direct evidence of EAF1-mediated enhanced ELL self-assembly, we used the purified proteins as shown in Supplementary Figure S4A in our *in vitro* self-assembly assay. As shown in Figure 5D, with suboptimal concentration of GST-ELL and GFP-ELL, we still observed their self-association (lane 3). However, consistent with our observation in cell-based assay, addition of purified EAF1 markedly enhanced the self-association between the ELL proteins (lane 4). Consistent with a role of EAF1 in enhancing ELL self-association, an EAF1 knockdown resulted in reduced ELL self-association within mammalian cells (Figure 5E, compare lane 2 versus lane 3).

For providing further mechanistic insights into these overall regulations, we initially checked the ELL domains that would be required for its interaction with EAF1 and SEC. As shown in Figure 5F, deletion of C-terminal occludin homology domain (501–621) completely abolished ELL interaction with both EAF1 and SEC (compare lane 2 versus lane 5). However, deletion of N-terminal 44 amino acids, that retained C-terminal occludin homology domain, showed strong reduction of ELL interaction with EAF1 without any effect on SEC interaction (Figure 5F, lane 6). In fact, we reproducibly observed enhanced SEC interaction with ELL upon deletion of N-terminal 44 amino acids (Figure 5F, lane 6 and Figure 5G, lane 3). Thus, these observations led us to conclude that a) for ELL interaction with EAF1, both the N and C-terminal domains are critical, b) SEC interaction is restricted within the C-terminal domain and c) a reduction of EAF1 association enhances ELL interaction with SEC components. Consistent with a role of N-terminal 44 amino acids of ELL in its interaction with EAF1, we have failed to observe any effect of addition of EAF1 on enhanced ELL self-assembly when 45–621 fragment of ELL is used in the self-assembly assay both *in vitro* (Figure 5H, compare lane 4 versus lanes 5–6) and cell-based assays (Supplementary Figure S5A, compare lane 4 versus lanes 5–6).

We next checked whether EAF2 protein, that interacts only with N-terminal end of ELL, would also show similar effect on ELL self-association. Interestingly, unlike EAF1, addition of EAF2 showed opposite effect and decreased ELL self-association in our cell-based assay (Figure 5I, compare lane 3 versus lane 4). However, our subsequent glycerol gradient-based fractionation of protein complex showed absence of EAF2 (like that of EAF1) in the ELL•SEC complex (Supplementary Figure S5B). Overall, these results thus imply that human ELL protein has an intrinsic ability to self-associate, that requires both N and C-terminal end, for its potential functional regulation (Supplementary Figure S5C). The EAF1 protein, by virtue of its interaction with both N and C-terminal ends, enhances ELL self-assembly through enhancing multivalent protein-protein interactions between ELL and EAF1, thus reducing ELL interaction with SEC components leading to transcriptional downregulation (Supplementary Figure S5C). EAF2 protein, on the other hand, interacts only with N-terminal end and thus blocks N and C-terminal-dependent ELL self-association. However, absence of EAF2 in the ELL•SEC further implies an unidentified function of EAF2 in regulation of ELL functions within mammalian cells. The strong effect of EAF1 on enhancing ELL self-association leading to reduced SEC association and transcriptional downregulation prompted us to further investigate the functional role of this phenomenon in regulating biological processes within mammalian cells.

### Exposure to genotoxic stress causes enhanced ELL self-association and its reduced association with SEC components

**Figure 6.**
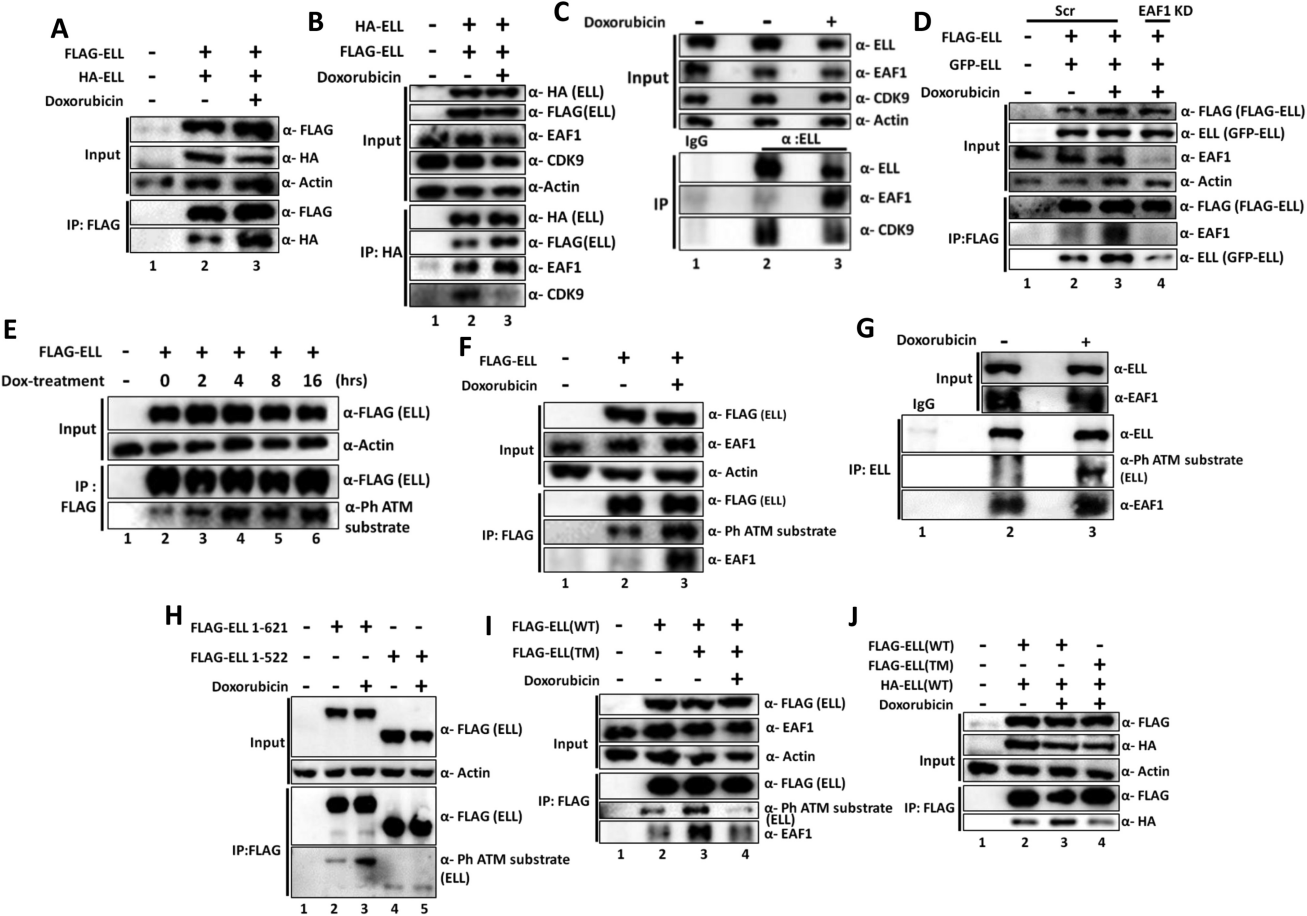
ATM-mediated ELL phosphorylation enhances EAF1 association and its self-association upon genotoxic stress. (**A**) Immunoblot analysis showing an increased self-association between ELL proteins in response to genotoxic stress within mammalian cells. Post 36 h of transfection with indicated plasmids, 293T cells were treated with doxorubicin for 2 hrs and the lysates were subjected to anti-FLAG immunoprecipitation followed by western blotting analysis using factor-specific antibodies as indicated. (**B**) Immunoblot analysis showing increased ELL self-association, EAF1 interaction and concomitant reduced SEC interaction (CDK9 as representative) within mammalian cells upon exposure to genotoxic stress. (**C**) Immunoblot analysis showing increased EAF1 and reduced SEC component (CDK9 as representative) association with endogenous ELL within mammalian cells upon doxorubicin treatment. (**D**) Immunoblot analysis showing defective genotoxic stress-induced ELL self-association in EAF1 knockdown cells. (**E**) Immunoblot analysis showing increased ATM-mediated phosphorylation of ectopically-expressed ELL within mammalian cells upon doxorubicin treatment. (**F**) Immunoblot analysis showing increased ATM-mediated phosphorylation of ELL and concomitant enhanced interaction with EAF1. (**G**) Immunoblot analysis showing genotoxic stress-induced increased ATM-mediated phosphorylation of endogenous ELL and concomitant enhanced EAF1 interaction within mammalian cells. (**H**) Immunoblot analysis showing presence of key ATM target sites at the C-terminus of ELL (521–621) which undergo phosphorylation in response to genotoxic stress. (**I**) Immunoblot analyses showing defective stress-induced ATM-mediated phosphorylation and EAF1 interaction with ELL triple mutant (TM, T551A, S561A, S589A). (**J**) Immunoblot analysis showing defective stress-induced self-association ability of ELL TM.

Temporal downregulation of transcription upon exposure to genotoxic stress is a key response for avoiding fatal collision between ongoing transcription and DNA repair machineries (2,3). Our earlier report has shown a role for p300-mediated acetylation of SEC component AFF1 in temporal regulation of transcription within mammalian cells (12). Since ELL is a *bona fide* elongation factor within SEC, we wondered whether ELL would also be subjected to temporal functional regulation for transcriptional inhibition upon exposure to genotoxic stress. We used doxorubicin that inhibits functions of topoisomerase II as well as intercalates within DNA resulting in DNA damage as indicated through enhanced phosphorylated H2AX at Ser-139 (γ-H2AX) signal (Supplementary Figure S6A) (35). Our initial analyses showed enhanced ELL self-assembly upon exposure to doxorubicin both by immunoprecipitation as well as cell-based colocalization studies (Figure 6A and Supplementary Figure S6B). This effect is not specific to doxorubicin only, since use of ionizing radiation (IR) also causes enhanced ELL self-assembly (Supplementary Figure S6C). Enhanced ELL self-assembly also accompanied with enhanced binding of EAF1 and concomitant reduced association with other SEC components such as CDK9 (Figure 6B, compare lane 2 versus lane 3). Immunoprecipitation of endogenous ELL using specific antibody, after exposure of cells with doxorubicin, showed enhanced association with EAF1 and concomitant reduced association with CDK9 (Figure 6C, compare lane 2 versus lane 3 in IP panel) and thus further indicated a role for EAF1 in enhancing this overall self-association (as has been observed earlier in our *in vitro* as well as cell-based studies as shown in Figure 5). Consistent with this hypothesis, we observed marked reduction of doxorubicin-induced enhanced ELL self-association upon EAF1 knockdown (Figure 6D, compare lane 3 versus lane 4). In fact, EAF1 knockdown resulted in even reduced self-association of ELL than that observed under normal cellular growth (Figure 6D, compare lane 2 versus lane 4). Based on all these observations, we conclude that EAF1-mediated enhanced ELL self-assembly, upon exposure to genotoxic stress, could be a key mechanism for temporal regulation of ELL functions for regulating global transcription.

### ATM-mediated ELL phosphorylation enhances EAF1 association upon exposure to genotoxic stress

For further mechanistic understanding into this genotoxic stress-induced temporal functional regulation of ELL, we initially addressed whether ELL would be subjected to ATM-mediated phosphorylation, a key response upon exposure to genotoxic stress. Interestingly, and consistent with our hypothesis, using an antibody that specifically recognizes ATM-mediated phosphorylation at S/T-Q sites, we observed enhanced phosphorylation of ectopically-expressed ELL upon exposure to doxorubicin with overall level of signal being increased with increasing incubation time (Figure 6E). Similar response is also observed after exposure to IR (Supplementary Figure S6D). This response is not transcription-dependent since prior treatment of cells with transcription blocking reagent DRB (a P-TEFb inhibitor) did not show much effect on genotoxic stress-induced ATM-mediated ELL phosphorylation (Supplementary Figure S6E, compare lane 3 versus lane 4). Enhanced ATM-mediated ELL phosphorylation is also accompanied with increased EAF1 association suggesting a role of this phosphorylation in overall functional regulation (Figure 6F, compare lane 2 versus lane 3). Immunoprecipitation of ELL using specific antibody further confirmed doxorubicin-induced ATM-mediated phosphorylation and enhanced EAF1 association with endogenous ELL as well (Figure 6G, compare lane 2 versus lane 3). This response is specific to ATM since usage an ATM inhibitor (ATMi, KU55933) markedly reduced ELL phosphorylation and corresponding enhanced EAF1 association (Supplementary Figure S6F, compare lane 3 versus lane 4).

To address the specific residues within ELL that are being targeted for ATM-mediated ELL phosphorylation upon genotoxic stress, we performed a domain analysis which showed that deletion of C-terminal 523–621 amino acids markedly reduced ELL phosphorylation upon doxorubicin treatment (Figure 6H, compare lane 3 versus lane 5). Interestingly, within this region of ELL, there are three S/T-Q sites (T551, S561 and S589) that could potentially be phosphorylated by ATM (Supplementary Figure S6G). Consistent with a role for these residues of ELL being phosphorylated by ATM, an earlier study reported ATM-mediated ELL phosphorylation at S561 residue by global highthroughput analysis (36). Introducing alanine mutations within these three residues, referred as triple mutant (TM, T551A, S561A and S589A), showed significantly reduced ATM-mediated phosphorylation suggesting that these residues are primarily targeted for ATM-mediated phosphorylation upon doxorubicin treatment (Figure 6I, compare lane 3 with lane 4). Reduced ATM-mediated phosphorylation of this ELL triple mutant also resulted in reduced EAF1 association (Figure 6I, compare lane 3 versus lane 4) and self-assembly upon exposure to doxorubicin (Figure 6J, compare lane 3 versus lane 4). Further, consistent with a role of ELL self-association and transcriptional activity, we observed enhanced ELL self-association during the early time points after IR treatment that coincided with transcriptional inhibition (Supplementary Figure S6H). However, at the later time points, during transcriptional recovery (see below), this self-association is reduced to basal level. These observations, thus suggest a critical role of dynamic regulation of ELL self-association in regulation of overall transcriptional activity within mammalian cells. Therefore, we conclude that, upon exposure to genotoxic stress, ATM-mediated phosphorylation of ELL in three key residues at the C-terminal end results in enhanced EAF1 association leading to enhanced self-assembly that could potentially be an important mechanism in temporal downregulation of transcription by ELL upon exposure to genotoxic stress.

### Genotoxic stress-induced enhanced EAF1 association with ELL is required for global transcriptional inhibition

**Figure 7.**
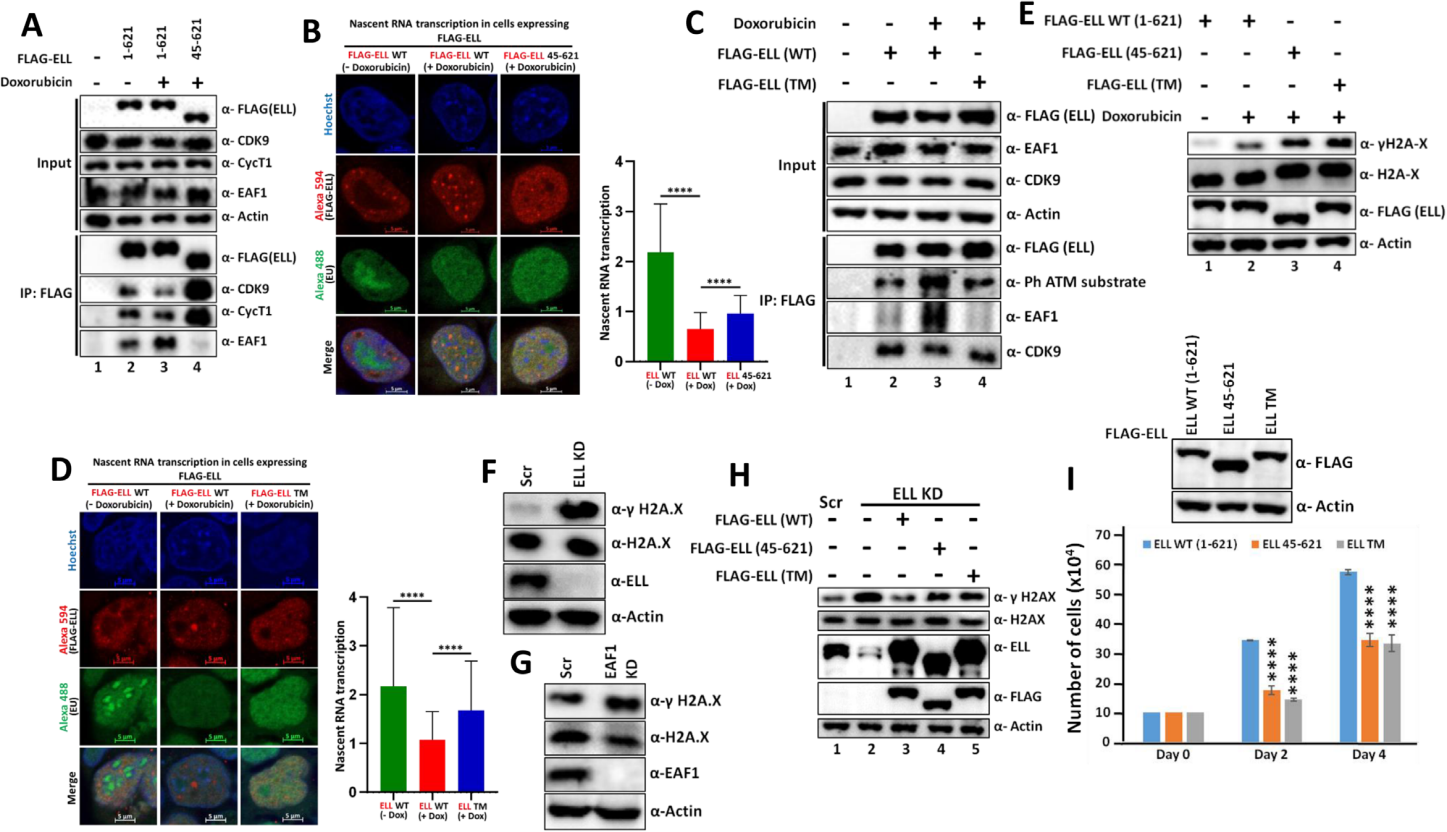
Self-association-defective ELL mutants fail to inhibit global transcription upon genotoxic stress and thus increases DNA damage and reduces cell survival. (**A**) Immunoblot analysis showing reduced ability of ELL (45–621) mutant, when compared to full-length, to interact with EAF1 and concomitant increase in other SEC component association (P-TEFb complex as representative) within mammalian cells upon exposure to genotoxic stress. (**B**) Nascent RNA transcription analysis showing enhanced global transcriptional activity in cells expressing ELL (45–621) mutant, when compared to full-length ELL upon exposure to genotoxic stress.293T cells were transfected with plasmids as indicated. 36hrs post transfection, cells were treated with or without doxorubicin for 2hrs as indicated. 15min prior to harvesting, EU was added for its incorporation into nascent RNA. These cells were then processed (as mentioned in the Methods section) for identifying overall global transcriptional activity as indicated. The data, as presented, represents normalization of signals obtained from green fluorescence (representing nascent RNA) over red fluorescence (representing ELL proteins). The right panel represents the quantification of the overall observation. (**C**) Immunoblot analysis showing reduced ATM mediated phosphorylation of ELL TM and concomitant decreased EAF1 interaction when compared to ELL WT within mammalian cells upon exposure to genotoxic stress. (**D**) Nascent RNA transcription analysis showing enhanced global transcriptional activity in cells expressing ELL TM, when compared to ELL WT upon exposure to genotoxic stress. Experiments were performed following the procedure as mentioned in (B). The data, as presented, represents normalization of signals obtained from green fluorescence (representing nascent RNA) over red fluorescence (representing ELL proteins). The right panel represents the quantification of the overall observation. In our nascent RNA transcription assays error bar represents mean ± SD, representative of two independent experiments (*n* = 80 cells). Statistical analysis was performed using two tailed *t*-test wherein, **** denotes *P* ≤ 0.0001. (**E**) Immunoblot analysis showing increased level of y-H2AX in cells expressing different ELL mutants (45–621 and TM) as compared to WT ELL within mammalian cells under genotoxic stress. (**F**) Immunoblot analysis showing increased level of DNA damage marker y-H2AX in ELL knockdown cells. (**G**) Immunoblot analysis showing increased level of DNA damage marker y-H2AX in EAF1 knockdown cells. (**H**) Immunoblot analysis showing the effect of re-expression of wild type ELL and its mutant derivatives in ELL knock down cells on the level of DNA damage marker y-H2AX. (**I**) Cell proliferation assay showing reduced proliferation ability of cells expressing self-association-defective ELL mutants as compared to WT after 2 h of doxorubicin treatment.

For further understanding of functional relevance of EAF1-induced enhanced ELL self-assembly in genotoxic stress-mediated transcriptional regulation, we initially used N-terminal deleted ELL mutant (45–621) that fully retained doxorubicin-induced ATM-mediated phosphorylation as well as SEC interaction (Supplementary Figure S7A and 7A respectively), however, fails to show EAF1-induced enhanced self-association (Figure 5). For understanding dynamic regulation of transcription by this ELL mutant upon exposure to genotoxic stress, we used 5-ethynyl uridine (EU, an uracil analog) incorporation assay for measuring nascent RNA transcription within cells at specific time point after doxorubicin treatment. Treatment with doxorubicin significantly decreased ongoing transcription in cells transfected with WT ELL (Figure 7B, images representing transcription on left panel and quantification on right panel). However, over-expression of 45–621 ELL mutant that fails to interact with EAF1 also failed to significantly reduce transcription suggesting enhanced transcriptional activity in the cells expressing this ELL mutant. Consistent with enhanced EAF1 association with ELL upon doxorubicin treatment for global transcriptional downregulation, the ELL TM that failed to show enhanced EAF1 association upon doxorubicin treatment (Figure 7C, compare lane 3 versus lane 4) also failed to reduce transcription as measured by nascent RNA transcription analysis (Figure 7D). Thus, these data clearly demonstrate a role for ATM-mediated phosphorylation of ELL resulting in enhanced EAF1 association as a key mechanism for genotoxic stress-induced global transcriptional downregulation.

### Global transcriptional downregulation through regulation of ELL activity is key for optimal DNA repair and cell survival upon genotoxic stress

Upon exposure to genotoxic stress, immediate global transcriptional downregulation is key for avoiding fatal collision between ongoing transcription and DNA repair machineries and providing proper access of damaged DNA to the repair machineries for optimal repair and survival of cells. Therefore, we initially tested whether the ELL mutants that failed to show optimal downregulation of transcription upon exposure to genotoxic stress, would also show enhanced DNA damage upon doxorubicin treatment. As shown in Figure 7E, in presence of ectopically-expressed WT ELL, we observed marked increase in γ-H2AX signal upon doxorubicin treatment (compare lane 1 versus lane 2). However, and importantly, over-expression of ELL mutants, that failed to optimally reduce transcription upon doxorubicin treatment, showed enhanced γ-H2AX signal than the wild type upon doxorubicin treatment (Figure 7E, compare lanes 3 and 4 versus lane 2). These results, thus indicate a critical role for ELL-mediated transcriptional downregulation in optimal DNA repair response. Interestingly, consistent with a role for functional regulation of EAF1-mediated ELL-dependent transcriptional downregulation in maintaining optimal genomic integrity, we have observed enhanced γ-H2AX signal in both ELL as well as EAF1 knockdown cells under normal growth condition itself (Figure 7F and G respectively). Whereas, re-expression of WT ELL in the ELL knockdown cells markedly reduced the overall γ-H2AX signal (Figure 7H, compare lane 2 versus lane 3), the ELL mutants that showed impaired EAF1 association and transcriptional downregulation, also failed to optimally reduce γ-H2AX signal (Figure 7H, compare lane 3 versus lanes 4–5). The enhanced presence of damaged DNA in the cells expressing defective ELL mutants also resulted in reduced cell survival after doxorubicin treatment (Figure 7I).

Thus our extensive biochemical and cell-based assays clearly show that upon exposure to genotoxic stress, mammalian cells employ the intrinsic self-association ability of key elongation factor ELL, for global transcriptional downregulation needed for optimal DNA repair response for maintaining genomic integrity and cell survival. Damage-dependent ATM-mediated phosphorylation enhances ELL association with EAF1 that further promotes the self-association and thus forming increased ELL•EAF1 complex through enhancing the overall valency of these associations leading to reduced interaction of ELL with other SEC components. These overall responses lead to quick and optimal global transcriptional inhibition for downstream efficient DNA repair response leading to cell survival during exposure to genotoxic stress.

## DISCUSSION

Global down-regulation of transcription is one of the key initial responses employed by mammalian cells during exposure to genotoxic stress. Rather than employing a few selected mechanisms for controlling transcriptional activity, it is quite conceivable that mammalian cells would employ multiple mechanisms in achieving rapid downregulation of transcription. Further, towards achieving this goal, key transcription factors would plausibly be primary targets for attaining a robust response. Towards this, ELL, being a direct regulator of transcription elongation by Pol II, would be a critical target for the overall regulation of transcription. In this regard, our analyses show involvement of ATM-mediated phosphorylation-dependent enhanced ELL interaction with EAF1 as a key response for global transcriptional downregulation within mammalian cells upon exposure to genotoxic stress. Further, our studies point towards a novel role of EAF1 in this process, wherein, EAF1-induced enhanced ELL self-assembly causes reduced interaction with other SEC components for transcriptional downregulation. Genotoxic stress-dependent enhanced association of EAF1 with ELL is key since ELL mutants that show reduced EAF1 interaction without losing interaction with other SEC components, fail to efficiently downregulate transcription leading to inefficient downstream repair of damaged DNA. An overall model for this mechanism of regulation is presented in Supplementary Figure S7B.

Self-association of proteins have been shown to play diverse roles in regulation of biological processes including transcription. In some cases, self-association has been shown to activate, whereas, in others to repress transcription. In our earlier study, we have shown that self-association of ZMYND8 that promotes its dimer formation, preferentially associates with P-TEFb complex and thus activates transcription, whereas the monomeric form associates with repressor NuRD complex to reduce transcription (22). In this context, through this study, we have clearly demonstrated an opposite role of ELL self-association and its effect on subsequent association with other SEC components and thus transcriptional regulation. In this context, ELL protein undergoes ATM-mediated phosphorylation-dependent enhanced association with EAF1 that further enhances self-association and thus favoring quick and efficient shutdown of transcription upon genotoxic stress for optimal DNA repair and cell survival.

Human SEC has been shown to positively regulate transcription of a vast number of target genes through regulation of elongation by Pol II (8,31). Among the SEC components, ELL is the only *bona fide* transcription elongation factor that directly stimulates Pol II-mediated transcription elongation (9,15). Further, ELL has also been shown to regulate transcription of snRNA genes through formation of the LEC in association with other components that are not part of SEC (24,25). Besides, several other studies have also indicated the presence of other ELL-containing complexes in regulation of transcription as well as DNA repair (13,37). Our *in vitro* reconstitution of defined protein complexes as well as the purification of protein complexes from mammalian cells clearly showed the presence of a separate ELL•EAF1 complex that does not co-reside with other SEC or LEC components. Interestingly, EAF1-association reduced ELL interaction with both SEC as well as LEC components and thus represents a separate complex within mammalian cells. This transient complex formation, and thus enhanced ELL self association upon interaction of EAF1, could be a direct mechanism for global downregulation of transcription. In this context, our reporter analysis as well as native target gene expression analyses showed a direct role of EAF1, and not of its homolog EAF2, in overall downregulation of ELL-mediated target gene expression (Figs. 2 and S2). EAF1 has been shown to directly stimulate ELL-mediated transcription elongation by Pol II in *in vitro* transcription assays using purified factors (9,14). Based on our results, it could be speculated that EAF1, upon enhancing self-association of ELL, could bring in more number of ELL proteins in close proximity of elongating Pol II in the *in vitro* studies and thus results in positive effect in overall transcriptional output in absence of other transcription factors. However, within cellular system, the overall transcriptional activity by the Pol II is also dependent on other elongation factors present within SEC. By virtue of EAF1 enhancing ELL self-association and thus reducing its association with other SEC components results in overall transcriptional downregulation as observed in our study. Whether, this newly described ELL•EAF1 complex would participate in distinct steps of transcription, independent of ELL•SEC function, would require significant detailed analysis. Future studies would be designed to address some of these critical questions in transcriptional regulation involving ELL-containing complexes including SEC, LEC and ELL•EAF1 as described in this study.

Does ATM target phosphorylation of transcriptionally-engaged ELL at the damage site for this overall regulation? Our results using transcriptional inhibitor (DRB) prior to exposing the cells to genotoxic stress, still show optimal ELL phosphorylation (Supplementary Figure S6E, lane 4). This clearly indicates that the overall ATM-mediated phosphorylation is independent of recognition of damaged DNA by the elongating Pol II. It could highly be possible that sequential signal that ultimately activates the ATM kinase, upon exposure to genotoxic stress, would lead to phosphorylation of entire ELL population irrespective of transcriptional engagement for attaining optimal transcriptional downregulation. Once phosphorylated, it triggers enhanced ELL self-assembly through increased interaction with EAF1 and thus prompting quick sequestration of monomeric ELL that associates with SEC components for transcriptional activation.

Interestingly, an earlier study has shown a role of the MLL-ELL fusion protein in dispersing ELL and EAF1 present within Cajal bodies in MLL-ELL leukemic cell lines (38). Based on understanding from our experimental results, it could be predicted that through delocalizing the ELL and EAF1 proteins from Cajal bodies, MLL-ELL fusion proteins could enhance the overall pool of ELL-containing SEC (ELL•SEC) for transcriptional upregulation of some key genes, such as *HOX* cluster genes, the expression of which are critically dependent on SEC activity and are important regulators of hematopoiesis (23). An in-depth understanding of the mechanism of transcriptional regulation involving MLL-ELL fusion protein in overall transcriptional regulation and its implication in leukemogenesis would be a subject for future endeavors for the mechanistic understanding of SEC-mediated transcriptional regulation and its implication in leukemic pathogenesis.

## DATA AVAILABILITY

All the data that were required for publishing this study are available with this paper. The raw western blotting and microscopy images that were used for making figures, as shown in this study, can be accessed through Mendeley database by clicking the link https://data.mendeley.com/datasets/k53sbjzjdp/1.

## Supplementary Material

gkac943_Supplemental_File
