# Correction to ‘ATM-mediated ELL phosphorylation enhances its self-association through increased EAF1 interaction and inhibits global transcription during genotoxic stress’

**DOI:** 10.1093/nar/gkad454

**Published:** 2023-05-17

*Nucleic Acids Research*, Volume 50, Issue 19, 28 October 2022, Pages 10995–11012, https://doi.org/10.1093/nar/gkac943

In the originally published version of this manuscript the affiliation ‘Academy of Scientific and Innovative Research (AcSIR), Ghaziabad, 201002, India’ was inadvertently omitted for author Debabrata Biswas.

This error has been corrected.

