# Supplementary material for: ATM-mediated ELL phosphorylation enhances its self-association through increased EAF1 interaction and inhibits global transcription during genotoxic stress
Source: Nucleic Acids Res. 2022 Oct 28;50(19):10995–1012. doi: 10.1093/nar/gkac943 (PMC9638944; doi:10.1093/nar/gkac943)
Supplement: gkac943_Supplemental_File [file gkac943_supplemental_file.pdf]

Supplemental Figures and Legends

Figure S1

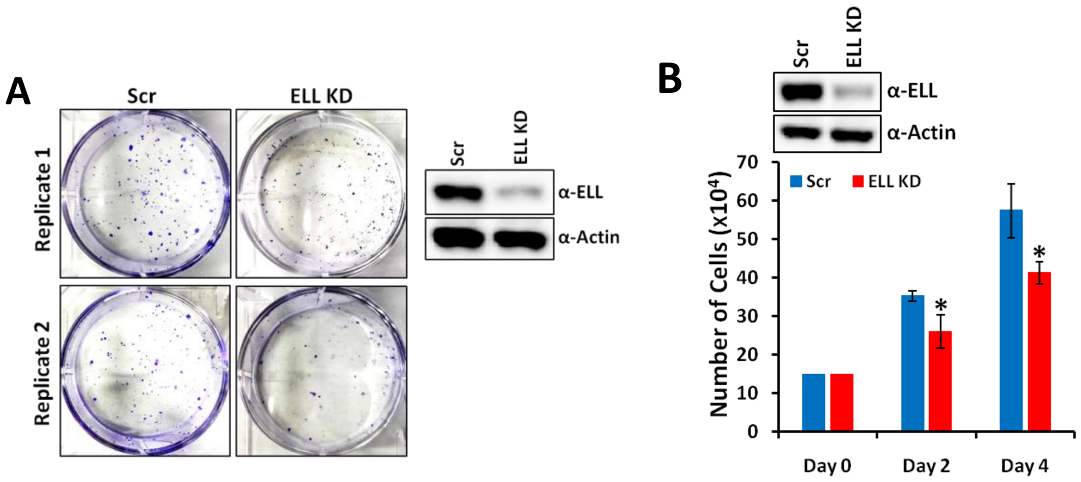

Figure S1: ELL knockdown causes reduced proliferation of mammalian 293T cells

A. Colony formation assay showing reduced colony forming ability of 293T cells upon stable knockdown of ELL. The inset western blots represent the efficiency of ELL knockdown by target shRNA.
B. Cell proliferation assay showing reduced proliferation ability of 293T cells upon stable knockdown of ELL. The inset western blots represent the efficiency of ELL knockdown by target shRNA.

The error bar in cell proliferation assay represents mean ± SD and statistical analyses were performed using one-tailed Student's *t* test wherein * denotes $p \leq 0.05$. The data represents quantifications from three independent experiments.

Figure S2

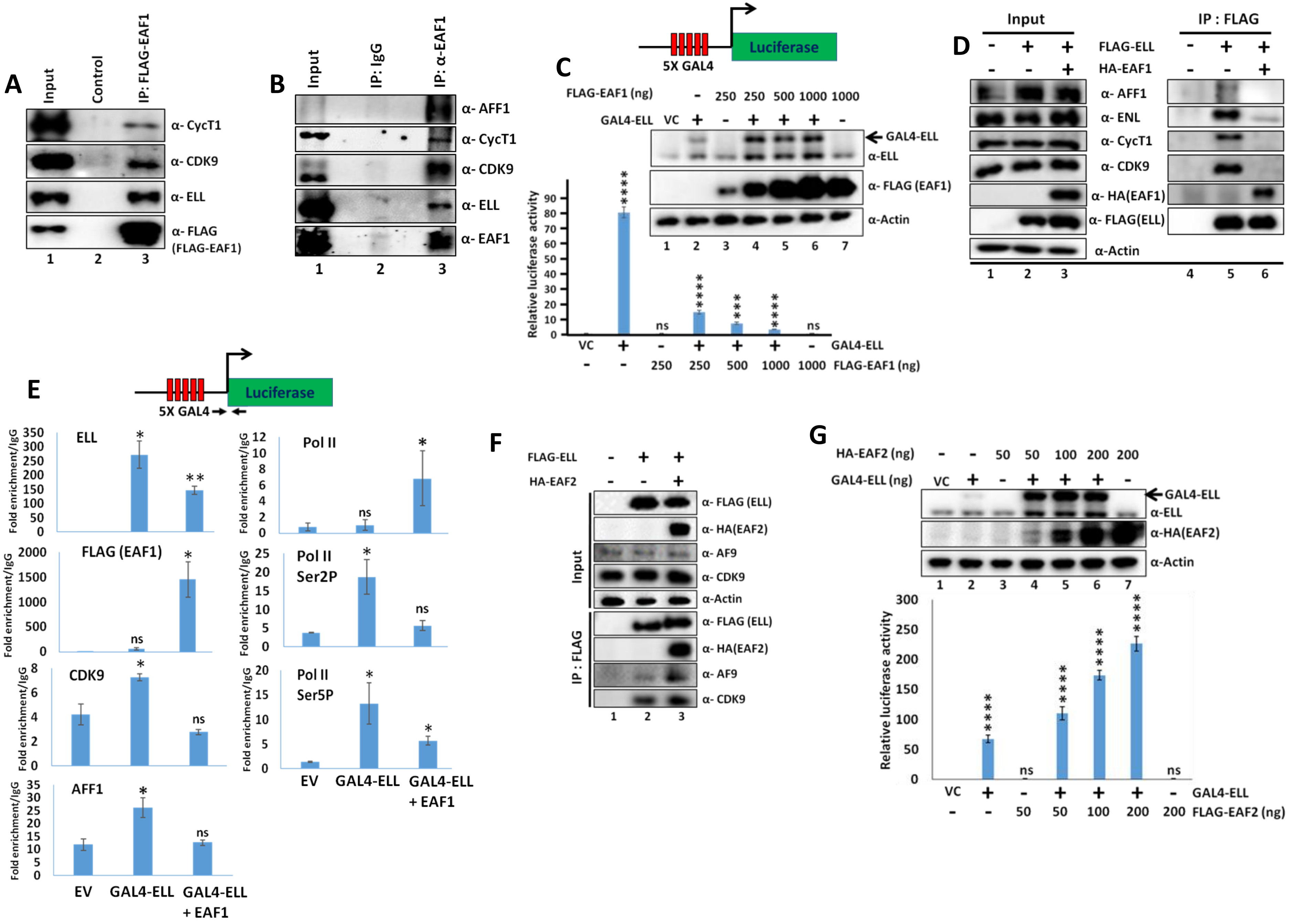

A
Input
Control
IP: FLAG-EAF1
α- CycT1
α- CDK9
α- ELL
α- FLAG (FLAG-EAF1)
1 2 3
B
Input
IP: IgG
IP: α-EAF1
α- AFF1
α- CycT1
α- CDK9
α- ELL
α- EAF1
1 2 3
C
5X GAL4
Luciferase
FLAG-EAF1 (ng) - 250 250 500 1000 1000
GAL4-ELL VC + - + + + -
GAL4-ELL
α-ELL
α- FLAG (EAF1)
α-Actin
1 2 3 4 5 6 7
Relative luciferase activity
ns
GAL4-ELL
FLAG-EAF1 (ng)
D
Input
IP : FLAG
FLAG-ELL
HA-EAF1
α- AFF1
α- ENL
α- CycT1
α- CDK9
α- HA(EAF1)
α- FLAG(ELL)
α-Actin
1 2 3 4 5 6
E
5X GAL4
Luciferase
Fold enrichment/IgG
ELL
FLAG (EAF1)
CDK9
AFF1
Pol II
Pol II Ser2P
Pol II Ser5P
ns
EV
GAL4-ELL
GAL4-ELL + EAF1
F
FLAG-ELL
HA-EAF2
Input
IP : FLAG
α- FLAG (ELL)
α- HA(EAF2)
α- AF9
α- CDK9
α-Actin
1 2 3
G
HA-EAF2 (ng) - - 50 50 100 200 200
GAL4-ELL (ng) VC + - + + + -
GAL4-ELL
α-ELL
α-HA(EAF2)
α-Actin
1 2 3 4 5 6 7
Relative luciferase activity
ns
GAL4-ELL
FLAG-EAF2 (ng)

Figure S2: Overexpression of EAF1, but not EAF2, reduces ELL-dependent expression of chromosomally-integrated reporter luciferase gene expression

A. Immunoblot analysis showing interaction between ectopically-expressed FLAG-EAF1 and different endogenous SEC components. 293T cells were transfected with plasmid expressing FLAG-tagged EAF1 and cell lysates were subjected to anti-FLAG immunoprecipitation. The immunoprecipitates were further analyzed by western blotting using factor-specific antibodies to identify interacting proteins as indicated.

B. Immunoprecipitation of endogenous EAF1 showing its interaction with other SEC components within mammalian cells. 293T cell lysates were immunoprecipitated using EAF1-specific antibody and IgG (as control). The immunoprecipitates were subsequently analyzed by western blotting using target factor-specific antibodies for identifying the interacting proteins as indicated.

C. Luciferase assay showing the negative effect of EAF1 on ELL-mediated reporter luciferase gene activation. Gal-luciferase 293T cells containing chromosomally-integrated reporter luciferase gene were co-transfected with GAL4-ELL and FLAG-EAF1 expressing plasmids as indicated and proceeded for luciferase activity analysis. Inset panel shows expression of respective proteins.

D. Immunoblot analysis showing the negative effect of EAF1 on association of ELL with other SEC components. 293T cells were co-transfected with indicated plasmids and cell lysates were subjected to anti-FLAG immunoprecipitation. The immunoprecipitates were further analyzed by western blotting using factor-specific antibodies to identify interacting proteins as indicated.

E. ChIP analysis showing the effect of EAF1 over-expression on the recruitment of SEC components, Pol II, and its phosphorylated Ser2 and Ser5 form at promoter proximal region as indicated of the target luciferase gene.

F. Immunoblot analysis showing the effect of EAF2 on association of ELL with other SEC components. 293T cells were co-transfected with indicated plasmids and cell lysates were subjected to anti-FLAG immunoprecipitation. The immunoprecipitates were further analyzed by western blotting using factor-specific antibodies to identify interacting proteins as indicated.

G. Luciferase assay showing the positive effect of EAF2 on ELL-mediated reporter luciferase gene activation. Gal-luciferase 293T cells containing chromosomally-integrated reporter luciferase gene were co-transfected with GAL4-ELL and HA-EAF2 expressing plasmids as indicated and proceeded for luciferase activity analysis. Inset panel shows expression of respective proteins.

The error bar in our luciferase and ChIP analyses represents mean ± SD and statistical analyses were performed using one/two tailed Student's t test wherein * denotes $p \leq 0.05$, ** denotes $p \leq 0.01$, *** denotes $p \leq 0.001$, **** denotes $p \leq 0.0001$ and ns denotes 'not significant'. The data represents quantifications from three independent experiments for luciferase assays and a minimum of n=2 biological replicates and three PCR replicates for each sample for ChIP assays.

Figure S3

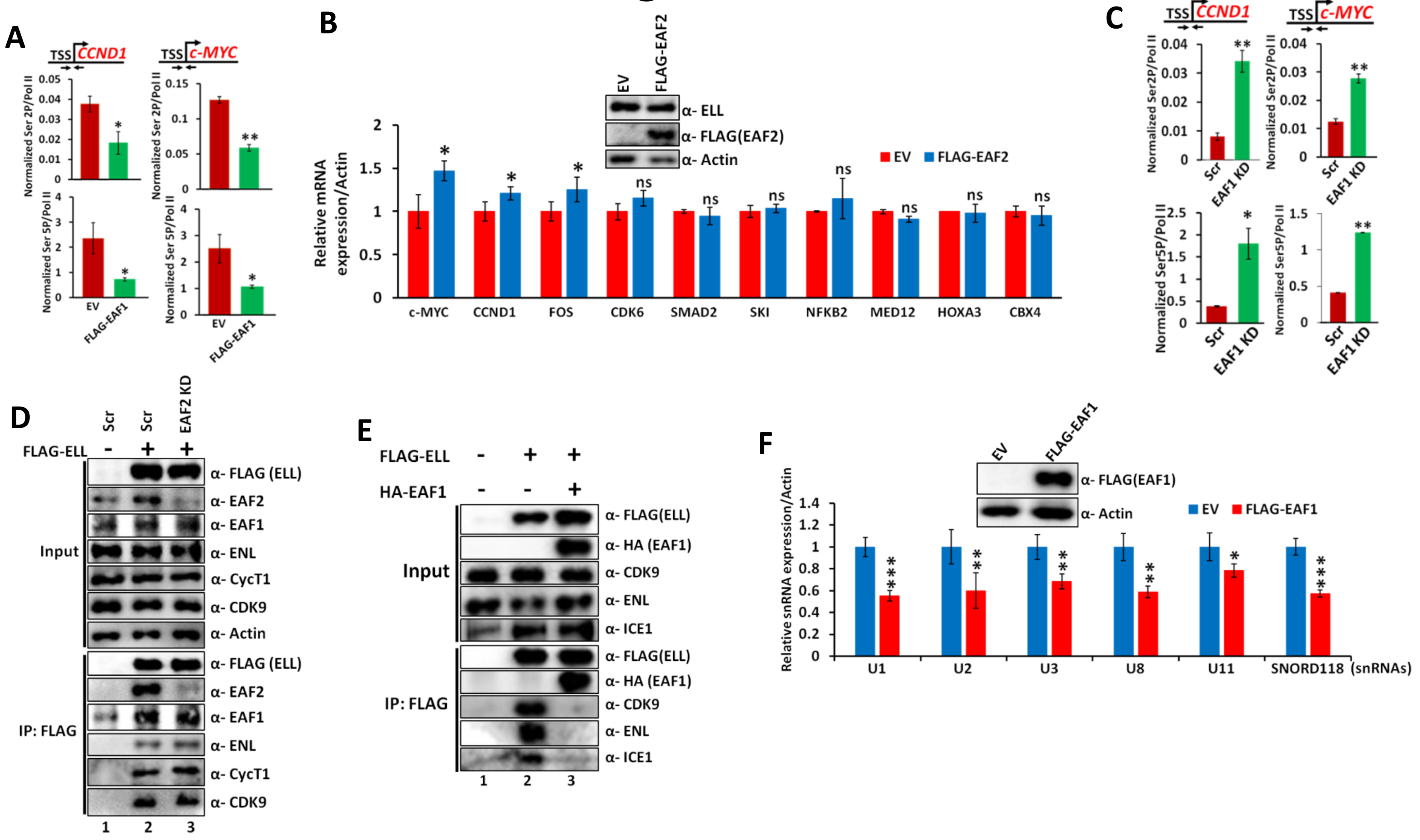

Figure S3: EAF2 fails to inhibit expression of ELL-dependent native target genes within mammalian cells.

A. ChIP analyses showing negative effect of EAF1 overexpression on the presence of Ser 2P and Ser 5P form of Pol II at the promoter proximal region of target *CCND1* and *c-MYC* genes (as shown in Fig. 2B). The fold enrichment of different factors on target region had been normalized with that of IgG control.

B. qRT-PCR analyses showing the effect of EAF2 overexpression on expression of ELL native target genes. 293T cells were transfected with plasmid constructs expressing empty vector (EV) and FLAG-EAF2 respectively. Total RNA was isolated post 48 hours of transfection and the expression of different target genes were analyzed by qRT-PCR.

C. ChIP analyses showing an enhanced presence of Ser 2P and Ser 5P form of Pol II at the promoter proximal region of target *CCND1* and *c-MYC* genes (as shown in Fig. 2E) upon EAF1 knockdown. The fold enrichment of different factors on target region had been normalized with that of IgG control.

D. Immunoblot analysis showing no significant effect of EAF2 knockdown on association of ELL with other SEC components within mammalian cells. Both scramble and EAF2 knockdown cells were transfected with plasmids expressing FLAG-ELL. Post 48 hours of transfection, cell lysates were subjected to anti-FLAG immunoprecipitation. The immunoprecipitates were further analyzed by western blotting using factor-specific antibodies to identify interacting proteins as indicated.

E. Immunoblot analysis showing the negative effect of EAF1 on association of ELL with other SEC as well as LEC components (ICE1). 293T cells were co-transfected with indicated plasmids and cell lysates were subjected to anti-FLAG immunoprecipitation. The immunoprecipitates were further analyzed by western blotting using factor-specific antibodies to identify interacting proteins as indicated.

F. qRT-PCR analyses showing the effect of EAF1 overexpression on expression of target snRNA genes within mammalian cells. 293T cells were transfected with plasmid constructs expressing empty vector (EV) and FLAG-EAF1 respectively. Total RNA was isolated post 48 hours of transfection and the expression of different target genes were analyzed by qRT-PCR.

In all of our qRT-PCR analyses for ChIP and RNA analyses, the error bar represents mean ± SD and statistical analyses were performed using one/two tailed Student's *t* test wherein * denotes $p \leq 0.05$, ** denotes $p \leq 0.01$, *** denotes $p \leq 0.001$, and ns denotes 'not significant'. Data represents a minimum of n=2 biological replicates and three PCR replicates for each sample.

Figure S4

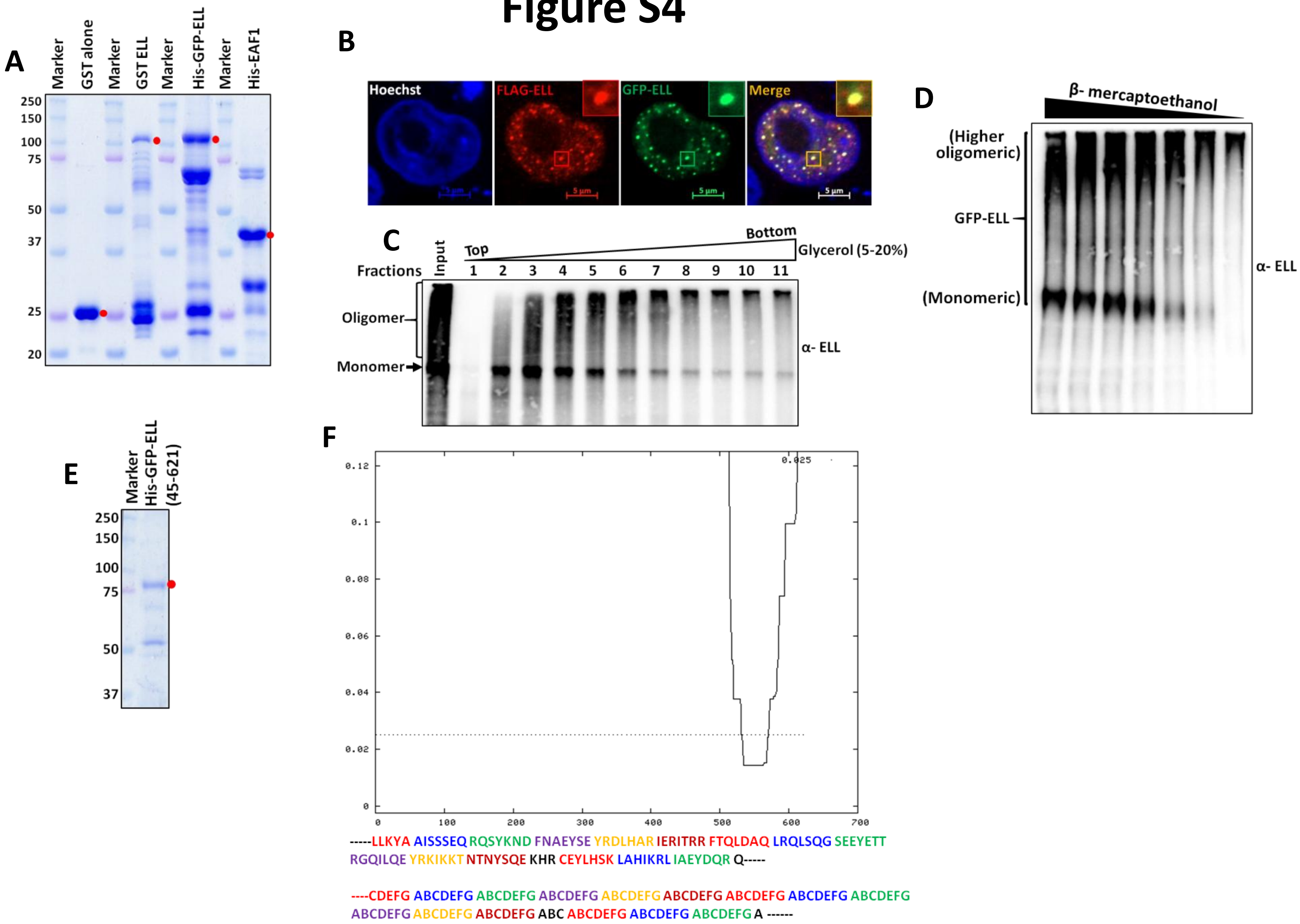

Figure S4: Purification of recombinant proteins and colocalization of ELL within mammalian cells

A. SDS-PAGE coomassie staining of purified recombinant proteins (as indicated) through their expression in bacterial expression system. Protein bands marked with red filled dots represent the target proteins in our assay and others are either non-specific or degradation of purified proteins.

B. Microscopic colocalization analysis showing the self-association between ELL proteins within the nucleus of mammalian 293T cells. 293T cells were transfected with plasmids expressing FLAG-ELL and GFP-ELL respectively. The cells were fixed and proceeded for immunofluorescence analysis post 48 hours of transfection.

C. Glycerol gradient-based separation of different self-associated ELL complexes as formed by purified recombinant His-GFP-ELL. Purified recombinant His-GFP-ELL was loaded onto a 5-20% glycerol gradient and was separated by centrifugation. Fractions were collected and individual fractions were tested for presence of different self-assembled ELL complexes by western blotting.

D. Immunoblotting analyses showing enhanced formation of higher oligomeric self-assembled ELL complex in reduced presence of β-mercaptoethanol.

E. Purification of recombinant N-terminal-deleted ELL fragment (45-621) through its expression in bacterial expression system.

F. Presence of coiled-coil domain at the C-terminal region of ELL as predicted by publicly available software paircoil2.

Figure S5

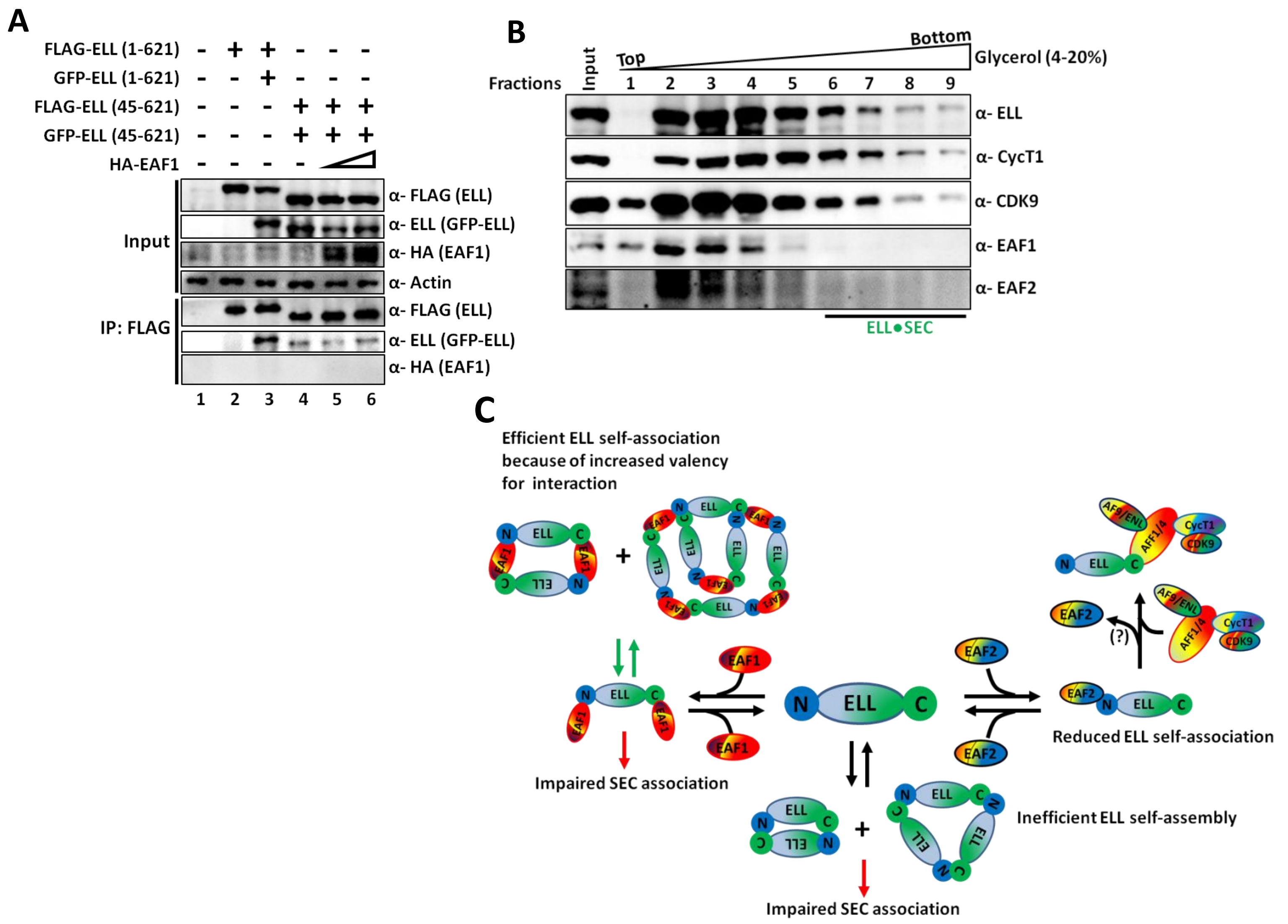

Figure S5: Model depicting the mechanism of EAF1-mediated enhanced ELL self-association and role of EAF2 in this process and their effect on ELL interaction with other SEC components

A. Immunoblot analysis showing failure of EAF1 protein to enhance self-association between N-terminal deleted (45-621) ELL fragments within mammalian 293T cells. 293T cells were transfected with indicated plasmids and cell lysates thus obtained were subjected to anti-FLAG immunoprecipitation. The immunoprecipitates were analyzed by western blotting using respective epitope tag-specific antibodies for identifying the effect of EAF1 on self-association.

B. Glycerol gradient-based separation of protein complex present in the nuclear extract of 293T cells showing absence of EAF2 in the ELL●SEC complex. Nuclear extract was loaded onto a 4-20% glycerol gradient and was separated by centrifugation. Fractions were collected and individual fractions were tested for presence of indicated proteins by western blotting.

C. Overall model depicting the differential role of EAF1 and EAF2 in regulation of ELL self-association and thus concomitant SEC association. The mechanism lies with the differential ability of EAF1 and EAF2 to interact with ELL on both the N and C terminus. As is clear from the domain analysis of ELL (from figures 4E and 5F) that, the SEC interaction with ELL is restricted to the C-terminal domain, but the self-association is dependent on both the N and C terminus of ELL. The EAF1 protein, by virtue of its interaction with both the N and C terminus increases ELL self-association through increasing the overall valency. However, interaction with EAF2 blocks the N-terminus of ELL and thus causes reduced ELL self-association and leads to increased SEC association and ELL-mediated target reporter gene activation (Fig. S2G). The increase in ELL self-association upon EAF1 interaction thus causes reduced SEC interaction and impaired ELL-mediated target gene activation (Figs. 2 and S2C).

Figure S6

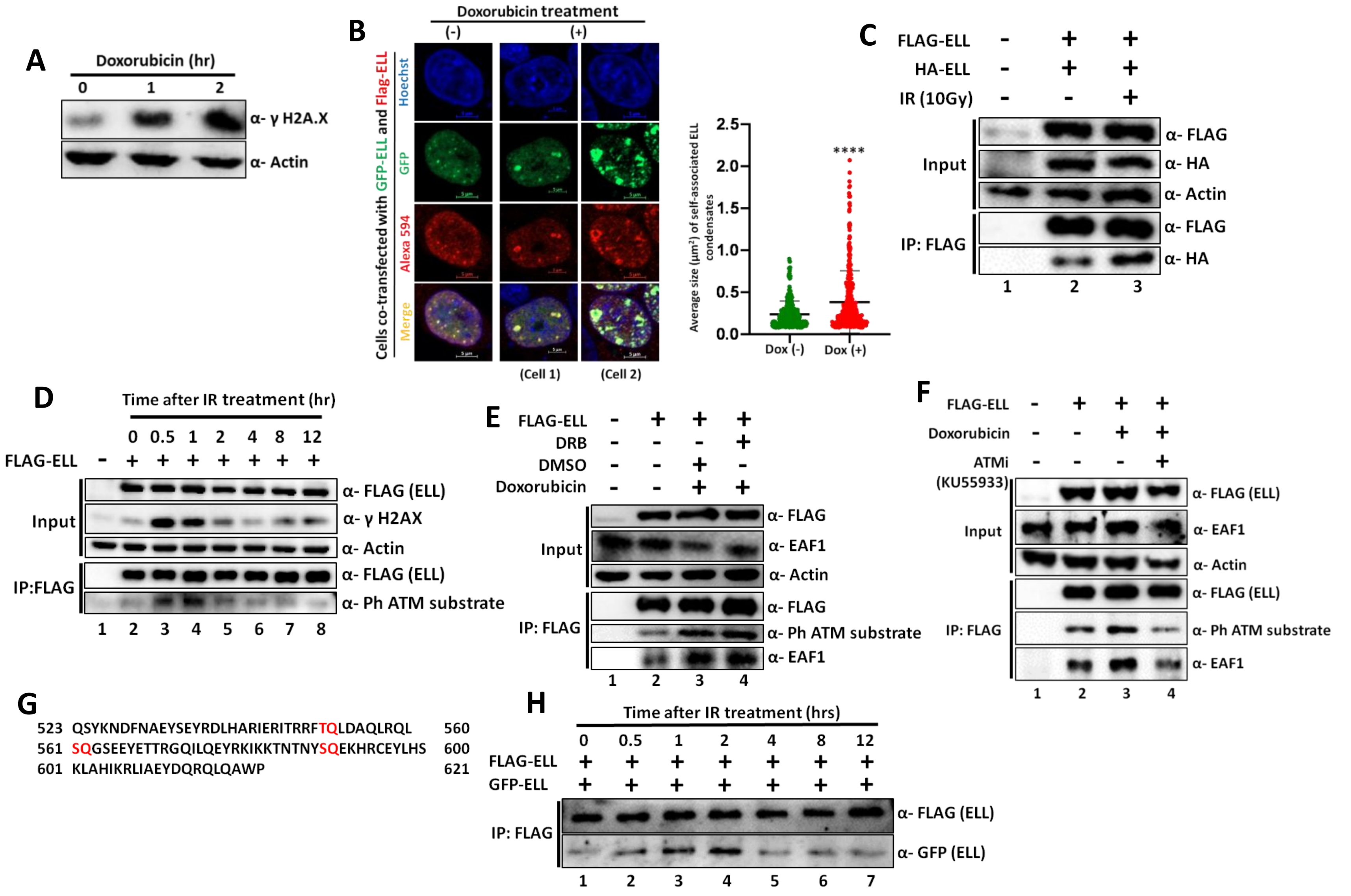

Figure S6: Genotoxic stress-induced enhanced ELL self-association is independent of type of stress and ongoing transcription

A. Immunoblot analysis showing increased levels of DNA damage marker γ-HA2X within mammalian cells in response to DNA damaging agent doxorubicin. 293T cells were treated with doxorubicin and harvested at indicated time points post treatment. The cell lysates thus obtained were subjected to western blotting analysis using factor-specific antibodies as indicated.

B. Microscopic immunofluorescence analysis using fixed cells showing enhanced self-association of ectopically-expressed ELL proteins within mammalian cells under genotoxic stress. 293T cells were transfected with plasmids expressing GFP- and FLAG-ELL and 36hrs post-transfection, cells were treated with or without doxorubicin as indicated and subsequently used for microscopic analysis. The Right panel represents the quantification of average size (μm^2) of self-associated puncta formed by ectopically-expressed ELL proteins. Error bar represents mean ± SD, n=350 colocalized puncta from >15 cells that have been imaged. Statistical analysis was performed using two tailed t- test wherein, **** denotes $p \leq 0.0001$.The data has been repeated three times with similar observations.

C. Immunoblot analysis showing increased self-association between ELL proteins in response to ionizing radiation (IR) treatment within mammalian cells. 293T cells were co-transfected with indicated plasmids and subjected to IR treatment post 36 hours of transfection. The lysates obtained from these cells were subsequently subjected to anti-FLAG immunoprecipitation. The immunoprecipitates were further subjected to western blotting analysis using factor-specific antibodies as indicated.

D. Immunoblot analysis showing IR-induced dynamic ATM-mediated phosphorylation of ectopically expressed ELL within mammalian cells. 293T cells were transfected with plasmid expressing FLAG-ELL and were subjected to ionizing radiation (IR) treatment as indicated. Cells were harvested at indicated time points post IR treatment. The cell lysates were then subjected to anti-FLAG immunoprecipitation. The immunoprecipitates were subjected to western blotting analysis using factor-specific antibodies as indicated.

E. Immunoblot analysis showing the effect of transcriptional inhibition on genotoxic stress-induced ATM-mediated ELL phosphorylation and EAF1 interaction. 293T cells were transfected with FLAG-ELL expressing plasmid. Post 36 hours of transfection, cells were treated with either DMSO or DRB (as indicated) for 30 minutes for inhibiting transcription prior to treatment with doxorubicin. The lysates obtained from these cells were subsequently subjected to anti-FLAG immunoprecipitation. The immunoprecipitates were subjected to western blotting analysis using factor-specific antibodies as indicated.

F. Immunoblot analysis showing the effect of ATM inhibitor (ATMi, KU55933) treatment on genotoxic stress-induced enhanced ATM mediated ELL phosphorylation and concomitant EAF1 interaction. Post 36 hours of transfection with indicated plasmids, 293T cells were pretreated with ATMi (KU55933) for 1 hr prior to doxorubicin treatment. The lysates were then subjected to anti-FLAG immunoprecipitation and western blotting analysis.

G. Potential ATM-mediated phosphorylation sites (S/T-Q) within 523-621 amino acid sequences of ELL that are being marked with red.

H. Immunoblot analysis showing IR-induced dynamic self-association of ectopically expressed ELL within mammalian cells. 293T cells were transfected with plasmid expressing FLAG-ELL and GFP-ELL were subjected to ionizing radiation (IR) treatment as indicated. Cells were harvested at indicated time points post IR treatment. The cell lysates were then subjected to anti-FLAG immunoprecipitation. The immunoprecipitates were subjected to western blotting analysis using factor-specific antibodies as indicated for identifying self-association between FLAG-ELL and GFP-ELL.

Figure S7

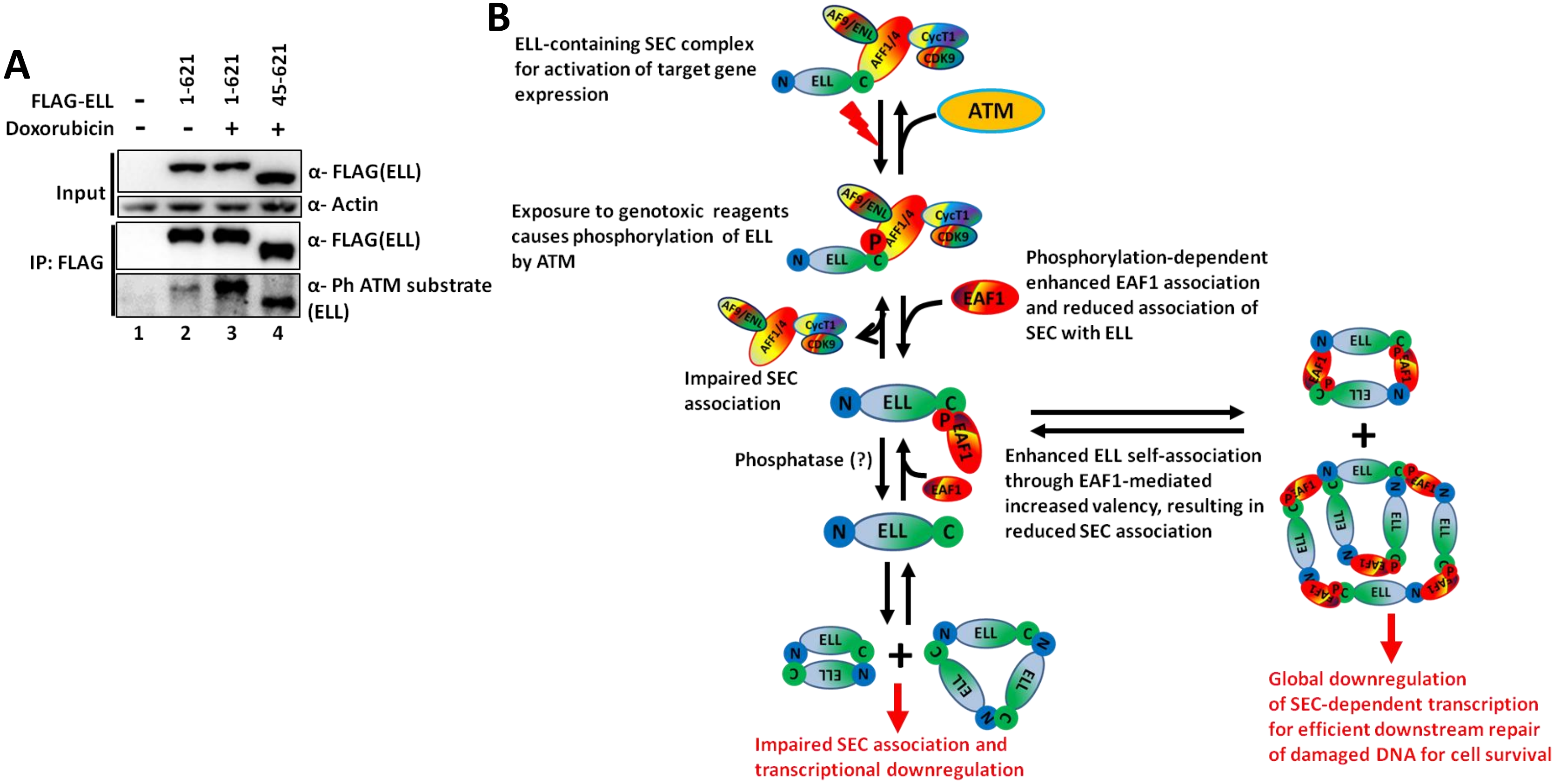

Figure S7: Overall model of EAF1-induced self-association of ELL in global transcriptional downregulation in response to genotoxic stress

A. Immunoblot analysis showing the ability of ELL 45-621 mutant to undergo genotoxic stress-induced ATM-mediated phosphorylation. 293T cells were transfected with plasmids expressing ELL WT and ELL 45-621, treated with doxorubicin as indicated. The lysates thus obtained were subjected to anti-FLAG immunoprecipitation. Immunoprecipitates were subsequently subjected to western blotting using factor-specific antibodies.

B. This model of transcriptional regulation is based on our observation of the absence of EAF1 in ELL-containing SEC. ELL protein, by virtue of its tendency to self-associate that requires both N and C terminus, the C-terminal end becomes inaccessible for its interaction with other SEC components. EAF1, by virtue of its ability to interact at both N and C terminus of ELL, provides additional valency for the ELL protein to interact with and further self-associate, thus enhancing overall self-association of ELL upon its binding and therefore causing transcriptional downregulation. Interestingly, genotoxic stress-dependent ATM-mediated ELL phosphorylation increases ELL-EAF1 interaction and thus leads to enhanced ELL self-association. This enhanced self-association further leads to reduced ELL binding with other SEC components and ultimately causes global transcriptional downregulation in response to genotoxic stress. This transcriptional downregulation is key for efficient downstream repair of damaged DNA, for overall cell survival upon exposure to genotoxic stress. ELL mutants with impaired ATM-mediated phosphorylation and EAF1 interaction, fail to efficiently downregulate global transcription upon exposure to genotoxic stress and leads to accumulation of DNA damage, causing reduced cell survival.

Supplemental Materials and Methods:

Supplemental Table 1: List of plasmids used in this study

Name	Description	Source
**M588**	ELL cloned into pM vector.	This study
**M455**	EAF1 cloned into 1X FLAG-tagged pcDNA5-FRT-TO plasmid.	This study
**M747**	ELL cloned into 1X FLAG-tagged pcDNA5-FRT-TO plasmid.	(1)
**M456**	EAF1 cloned into 1X HA-tagged pcDNA5-FRT-TO plasmid.	This study
**M459**	EAF2 cloned into 1X HA-tagged pcDNA5-FRT-TO plasmid.	This study
**M458**	EAF2 cloned into 1X FLAG-tagged pcDNA5-FRT-TO plasmid.	This study
**M1032**	ELL (1-572) cloned into 1X FLAG-tagged pcDNA5-FRT-TO plasmid.	This study
**M1033**	ELL (1-522) cloned into 1X FLAG-tagged pcDNA5-FRT-TO plasmid.	This study
**M742**	ELL (1-500) cloned into 1X FLAG-tagged pcDNA5-FRT-TO plasmid.	(1)
**M744**	ELL (45-621) cloned into 1X FLAG-tagged pcDNA5-FRT-TO plasmid.	(1)
**M621**	CDK9 cloned into 6X HIS-tagged pcDNA5-FRT-TO plasmid.	This study
**M49**	ELL cloned into FLAG-pFASTBAC vector.	(2)
**M412**	CDK9 cloned into 6X HIS-tagged pFASTBAC vector.	(3)
**M97**	AF4 cloned into NT-pFASTBAC vector.	This study
**M399**	Cyclin-T1 cloned into NT- pFASTBAC vector.	(3)
**M81**	AF9 cloned into NT-pFASTBAC vector.	(2)
**M74**	EAF1 cloned into NT-pFASTBAC vector.	(2)
**M141**	ELL cloned into 6X HIS-tagged pFASTBAC vector.	This study
**M94**	EAF1 cloned into FLAG-tagged pFASTBAC vector.	This study
**M411**	CDK9 cloned into NT-pFASTBAC vector.	This study
**M75**	EAF2 cloned into NT-pFASTBAC vector.	This study
**M155**	AF9 cloned into 6X HIS-tagged pFASTBAC vector.	(2)
**M1048**	ELL cloned into 1X HA-tagged pcDNA5-FRT-TO plasmid.	This study
**M56**	pET11d-GST vector.	(2)
**M61**	ELL cloned into pET-GST vector.	(2)
**M1156**	ELL cloned into 6X HIS-GFP- pET-11d vector.	This study
**M1273**	ELL (45-621) cloned into 6X HIS-GFP- pET-11d vector.	This study
**M498**	ELL cloned into pEGFP-N2 vector.	(1)
**M1200**	ELL TM (T551A,S561A,S589A) cloned into 1X FLAG-tagged pcDNA5-FRT-TO plasmid.	This study
**M1195**	ELL TM (T551A,S561A,S589A) cloned into pEGFP-N2 vector.	This study
**M1175**	ELL (45-621) cloned into pEGFP-N2 vector.	This study
**M447**	1X FLAG-tagged pcDNA5-FRT-TO plasmid.	(2)

Supplemental Table 2: List of qRT-PCR primer Sequences used for RNA analysis

Target gene	5' forward primer sequence	3' reverse primer sequence
**ELL**	GACCAACACCAACTACAGCCAGG	GTACTCGGCGATGAGCCTCTTG

**c-MYC**	GCTTGTACCTGCAGGATC	GACTCCGTCGAGGAGAG
**CCND1**	TCTAAGATGAAGGAGACCAT	GGAAGTGTTCAATGAAATCG
**CDK6**	GGAGTGTTGGCTGCATATTTG	CGATATCTGTTACAAACTTC
**NFKB2**	GAGAACGGAGACACACC	CAGAAAGCTCACCACAC
**FOS**	GGAACAGTTATCTCCAGAAG	ACTCTAGTTTTTCCTTCTCC
**SKI**	CCAAGTACTCGGCCCAGATC	GCACGGAATCTACGGCTCC
**MED15**	GACCTTTAAACACACCTGTG	GGCTCTTCATCTTACTCAGG
**BCL6**	CCACACAGGAGAGAAACCTTAC	GCAGGTCAGTGGCTGACAC
**HOXA3**	GAGTTCCACTTCAACCGC	CGATGACGTTAGCATGCC
**APOE**	GCGGATGGAGGAGATG	CTCGAACCAGCTCTTG
**TAF6**	CTGGGAGTGTCCAGAAG	CATTGGCTTTTGGAGTCC
**AF9**	GTAGAGCTTCACAGAAGG	GTAGTTTACGGACTGTGG
**CBX4**	CTGGTCGCCCAAATATAAC	TCAGGACATTGGAACGAC
**KIF1B**	AATCAGAGTGACTTTTCGTC	ATTCGACTGATTTCTTCTGG
**VDAC1**	CAAGTATCAGATTGACCCTG	GTCAGTTTAATACCTGGC
**SMAD2**	GCAGAGCCCCAATTGTAATC	GGTGCACATTCTAGTTAGCTG
**MED12**	CAGGATGAACAACGCGAG	GAGTGCCTCATGCATAAGC
**CDKN2B**	GGAGCAGCATGGAGCCTTC	GCTGCCCATCATCATGAC
**KLF5**	CCAGTATATTCAGCTCACACC	CCATTGCTGCTGTCTGATTTG
**PCNA**	GGAGGAAGCTGTTACCATAGAG	CCTCGATCTTGGGAGCCAAG
**EAF1**	CGTTGCCAATGGAACCAGCC	CATCACTGTCACTGCCAGACTC
**EAF2**	GCATCTCCAATAGATGATATCG	GTATCAGAAGTAGAGGATTTGC
**ACTIN**	AGAGCTACGAGCTGCCTGAC	AGCACTGTGTTGGCGTACAG
**U1 snRNA**	GGGAGATACCATGATCACGAAGGT	ATGCAGTCGAGTTTCCCACA
**U2 snRNA**	GTTTAATATCTGATACGTCCTCTATCC	TCGATGCGTGGAGTGGAC
**U3 snRNA**	GAGAAGTTTCTCTGAACGTGT	TCAATGGCTGACGGCAGTTG
**U8 snRNA**	GGGATAATCCTTACCTGTTCC	TCAGGGTGTTGCAAGTCCTG
**U11 snRNA**	TTCTGTCGTGAGTGGCACACGTA	AACGATCACCAGCTGCCCAAATAC
**SNORD118 snRNA**	GAGGGCAGATTAGAACATGATGA	GCAATCAGGGTGTTGCAAGT

Supplemental Table 3: List of qRT-PCR primer Sequences used for ChIP analysis

Target gene	5′ forward primer sequence	3′ reverse primer sequence
**c-MYC TSS**	TCCTCTCTCGCTAATCTCCGC	GGGTCCTCAGCCGTCCAGAC
**CCND1 TSS**	CGGGCTTTGATCTTTGCTTA	CTGCTGCTCGCTGCTACT
**c-MYC (+3 kb)**	GACTCTGGTAAGCGAAGC	TCCAGATCTGCTATCTCTCC
**CCND1 (+3 kb)**	CTCCTTAGGTGACCCTGG	GACAAACAATCCAGGGCCT
**Gal4-Luc TSS**	CTTATGGTACTGTAACTGAGCTAAC	GCGGGACTATGGTTGCTGAC

Supplemental Table 4: List of oligo-sequences used for generating shRNA constructs

Target genes	Upper oligo	Lower oligo
**ELL**	CCGGGCTACAAGAACGACTTCAATGCTCGAGCATTGAAGTCGTTCTTGTAGCTTTTTG	AATTCAAAAAGCTACAAGAACGACTTCAATGCTCGAGCATTGAAGTCGTTCTTGTAGC
**EAF1**	CCGGATCATGACACTGGTGAATATGCTCGAGCATATTCACCAGTGTCATGATTTTTTG	AATTCAAAAAATCATGACACTGGTGAATATGCTCGAGCATATTCACCAGTGTCATGAT

Supplemental Table 5: List of antibodies used in this study

Target proteins	Source	Catalogue no.
**ELL**	Cell Signaling Technology	14468S
**FLAG-epitope**	Sigma	F7425
**β-Actin**	Santa Cruz Biotechnology	sc-47778, BB-AB0024
**HA-epitope**	Santa Cruz Biotechnology	sc-57592
**AF9**	Bethyl Laboratories	A300-596
**CDK9**	Santa Cruz Biotechnology and Cell Signaling Technology	sc-8338, 2316.
**Cyclin-T1**	Santa Cruz Biotechnology	sc-10750
**ENL**	Cell Signaling Technology	14893
**AFF1**	Abcam	ab31812
**AFF4**	Gift from the Roeder lab	
**EAF1**	Santa Cruz Biotechnology	sc-373832
**EAF2**	Bethyl Laboratories	A302-503A
**GFP**	BioBharati Life Science	BB-AB0065
**GST**	Santa Cruz Biotechnology	sc-53909
**Phospho-ATM/ATR Substrate**	Cell Signaling Technology	9607S
**Rpb1 CTD**	Cell Signaling Technology	2629
**Phospho-Rpb1 CTD (Ser2)**	Cell Signaling Technology	13499

**Phospho-Rpb1 CTD (Ser5)**	Cell Signaling Technology	13523
**Mouse IgG**	Cell Signaling Technology	5415S
**Rabbit IgG**	Cell Signaling Technology	2729S

References:

1. Basu, S., Barad, M., Yadav, D., Nandy, A., Mukherjee, B., Sarkar, J., Chakrabarti, P., Mukhopadhyay, S. and Biswas, D. (2020) DBC1, p300, HDAC3, and Siah1 coordinately regulate ELL stability and function for expression of its target genes. *Proc Natl Acad Sci U S A*, **117**, 6509-6520.
2. Yadav, D., Ghosh, K., Basu, S., Roeder, R.G. and Biswas, D. (2019) Multivalent Role of Human TFIID in Recruiting Elongation Components at the Promoter-Proximal Region for Transcriptional Control. *Cell Rep*, **26**, 1303-1317 e1307.
3. Ghosh, K., Tang, M., Kumari, N., Nandy, A., Basu, S., Mall, D.P., Rai, K. and Biswas, D. (2018) Positive Regulation of Transcription by Human ZMYND8 through Its Association with P-TEFb Complex. *Cell Rep*, **24**, 2141-2154 e2146.
